# Metagenomic mapping of cyanobacteria and potential cyanotoxin producing taxa in large rivers of the United States

**DOI:** 10.1038/s41598-023-29037-6

**Published:** 2023-02-16

**Authors:** David M. Linz, Nathan Sienkiewicz, Ian Struewing, Erin A. Stelzer, Jennifer L. Graham, Jingrang Lu

**Affiliations:** 1 Office of Research and Development, U.S. Environmental Protection Agency, Cincinnati, OH USA; 2 U.S. Geological Survey, Columbus, OH USA; 3 U.S. Geological Survey, Troy, NY USA

**Keywords:** Freshwater ecology, Metagenomics, Microbial ecology

## Abstract

Cyanobacteria and cyanotoxin producing cyanobacterial blooms are a trending focus of current research. Many studies focus on bloom events in lentic environments such as lakes or ponds. Comparatively few studies have explored lotic environments and fewer still have examined the cyanobacterial communities and potential cyanotoxin producers during ambient, non-bloom conditions. Here we used a metagenomics-based approach to profile non-bloom microbial communities and cyanobacteria in 12 major U.S. rivers at multiple time points during the summer months of 2019. Our data show that U.S. rivers possess microbial communities that are taxonomically rich, yet largely consistent across geographic location and time. Within these communities, cyanobacteria often comprise significant portions and frequently include multiple species with known cyanotoxin producing strains. We further characterized these potential cyanotoxin producing taxa by deep sequencing amplicons of the *microcystin E* (*mcyE*) gene. We found that rivers containing the highest levels of potential cyanotoxin producing cyanobacteria consistently possess taxa with the genetic potential for cyanotoxin production and that, among these taxa, the predominant genus of origin for the *mcyE* gene is *Microcystis*. Combined, these data provide a unique perspective on cyanobacteria and potential cyanotoxin producing taxa that exist in large rivers across the U.S. and can be used to better understand the ambient conditions that may precede bloom events in lotic freshwater ecosystems.

## Introduction

Cyanobacteria and cyanotoxins are capable of rapid and dramatic increases in abundance, causing phenomena referred to as ‘harmful algal blooms’ (HABs). HABs can have significant impacts on human, ecological and economic health^1–4^. The frequency of HABs caused by cyanobacteria is predicted to grow with the increasing eutrophication of water ecosystems and global temperature rise^5,6^, although there is evidence that indicates climate change and temperature rise may not be as impactful as initially proposed^7^. HABs are complex phenomena and understanding the dynamics of cyanobacteria and cyanotoxins remains a significant challenge^8^. As such, it is critical to deepen our understanding of: (i) the cyanobacterial communities and cyanotoxin producing taxa present before, during, and after blooms, (ii) the non-cyanobacterial microbial communities that accompany cyanobacteria during these times, (iii) the fluctuation of these communities based on location and date, and (iv) how abiotic environmental factors can affect each of these community dynamics. Taken together, this knowledge will lead to a better understanding of cyanobacterial communities and the variables that can be used to understand, predict, and mitigate the effects of their occurrence.

Currently, many studies examining cyanobacteria and cyanotoxin production focus on lentic environments such as lakes, ponds, and reservoirs^9–16^. Further, many of these studies are conducted during cyanobacterial blooms, which often have few or even single taxa dominating a given environmental niche, reducing community diversity. While these studies are critical to our overall understanding, cyanobacteria present in exoreic lake and/or pond aquatic ecosystems are not isolated to these environments. Instead, they are connected to rivers and streams within their respective watersheds^17–24^. This interconnectedness means that rivers and their microbial communities are highly dynamic; a churning intermixing of their own ecosystems as well as those ecosystems feeding them (or into which they are draining). Additionally, rivers possess a varying conglomerate of physical, chemical, and other abiotic factors that can further impact microbial community dynamics including cyanobacteria and cyanotoxin producing species. Many metagenomic studies have been conducted on general river microbial communities (for examples see^25–36^). In contrast, considerably fewer studies have examined cyanotoxin producing cyanobacteria in rivers, and oftentimes these studies are limited to exploring only cyanotoxin synthetase genes from a handful of species^37–40^. Even fewer studies have profiled a river’s cyanobacterial communities using metagenomics-based approaches in the absence of a priori assumptions generated from known or reported increases in cyanobacteria and/or cyanotoxins^41^.

**Figure 1:**
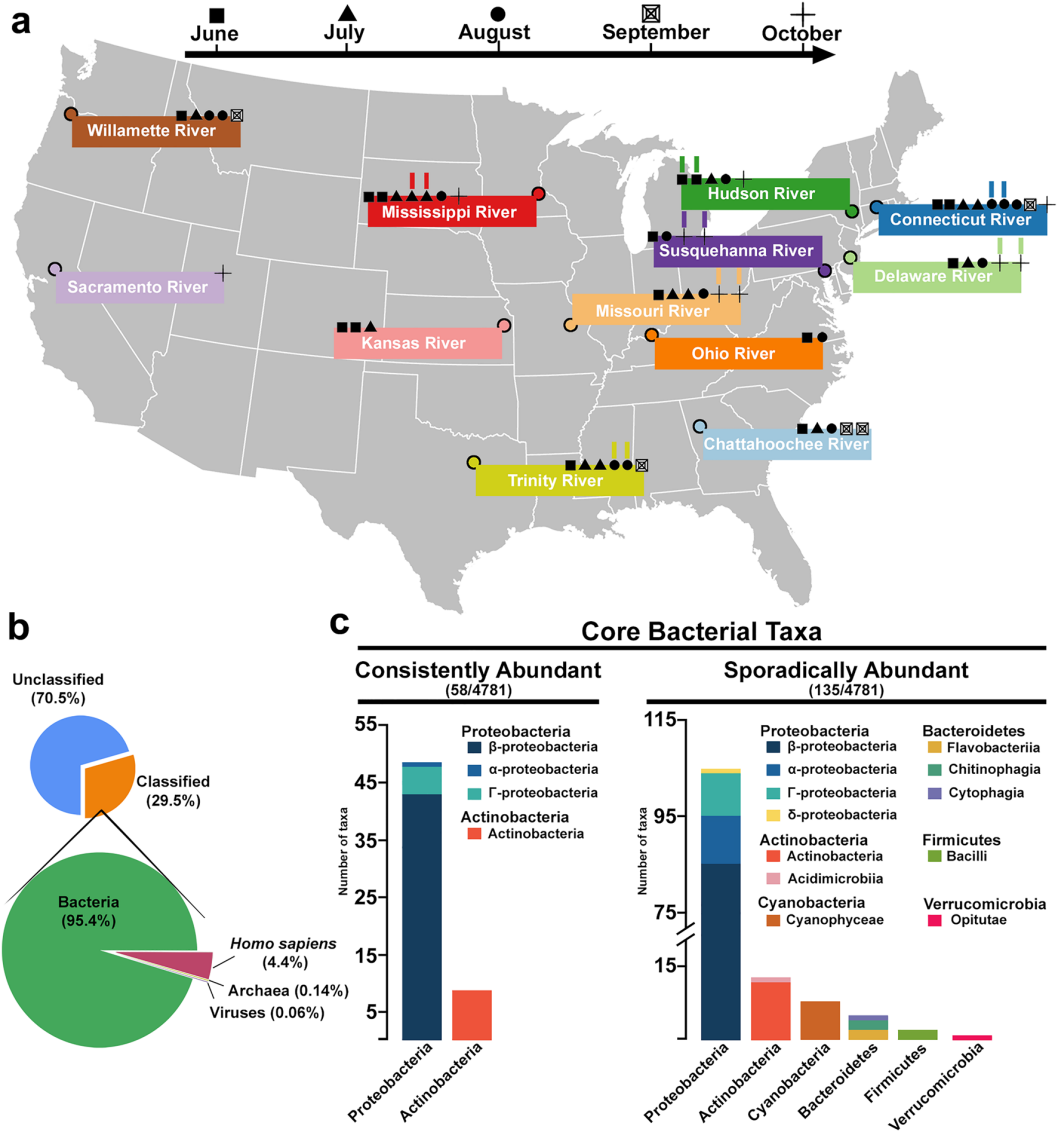
U.S. river samples and ‘core’ microbial communities. (**a**) River location and month of sampling (in 2019). Samples taken sequentially (replicates) are indicated by colored hashes above date shape. (**b**) Pie charts depicting reads that were unclassified (blue) vs classified (orange) (top pie chart) and their broad taxonomic distribution (bottom pie chart). (**c**) The taxonomic composition of the most abundant (core) river taxa at the phylum and class level. The left graph depicts taxa that were consistently highly abundant (> 0.1% relative abundance) across a majority of river samples (> 80%), while the right graph shows taxa that were highly abundant, but not consistently: taxa that were sporadically abundant.

During 2010–2017, the United States Geological Survey (USGS) conducted a study to better understand the occurrence of cyanobacteria with known cyanotoxin-producing strains, cyanotoxin synthetase genes, and cyanotoxins in lotic ecosystems^38,39^. To supplement that study, we sought to use a metagenomics-based approach to profile microbial communities within twelve large United States (U.S.) rivers from June to October in 2019 (Fig. 1a). The selected rivers are geographically distributed across the continental U.S. (Fig. 1a) and were chosen because they are part of the USGS network of long-term monitoring sites allowing paired physicochemical and environmental data for each location and date. Importantly, during the times samples were collected, no river was reported to be undergoing a substantial cyanobacterial bloom. Therefore, observed microbial and cyanobacterial community composition represent ambient, non-bloom conditions. We performed shotgun metagenomic sequencing and explored the microbial communities and, more specifically, the cyanobacterial communities within each river. We then searched for cyanotoxin producing cyanobacterial species and addressed how physicochemical and environmental variables were associated with these communities across the varying river ecosystems. Lastly, we amplified and deep sequenced a region of the *microcystin E* (*mcyE*) gene. *mcyE* is one of ten genes critical to the biosynthesis of the cyanotoxin microcystin (MC) and is highly variable amongst the many potential MC producing species allowing us to understand which cyanobacteria could produce MC and their proportional abundance within each river and time point^42–44^. These data also provided perspective on the genotypic diversity present within the amplified *mcyE* loci and how that diversity may be distributed across space and time. Combined, our data show that U.S. rivers possess taxonomically rich, yet broadly similar microbial communities that frequently contain significant cyanobacterial abundance. These cyanobacteria often contain potential cyanotoxin producing strains; the most dominant of which are MC producers that often have the genetic potential to produce toxin. These results represent a first attempt at summarizing the cyanobacteria present in the diverse lotic freshwater ecosystems of major continental U.S. rivers.

## Results

### Overall community structure and composition

We sampled rivers at various locations and dates across the U.S. in 2019 (Fig. 1a, Table S1). Our 300 base-pair (bp) paired-end Illumina MiSeq runs resulted in a final average of 1.9 × 10^6^ paired reads per sample. Of these, an average of 92% passed quality trimming and were subsequently classified for community profiling. One advantage of using shotgun metagenomics is that we are provided with a glimpse of overall community structure outside of purely bacterial taxa; however, because the Kraken2 database used for taxonomic classification does not comprehensively profile eukaryotic organisms, we cannot differentiate between those reads that remain truly unclassified and those that fall into the eukaryotes (barring one eukaryote we purposely included: *Homo sapiens*). Of the reads passed through the Kraken2 classifier, on average ~ 70% (57–80%) were either unable to be classified or belonged to the eukaryotes. Among the remaining classified reads (20–40%), an average of 95% (88–99%) were classified as bacteria, 4% (0.6–11%) as *Homo sapiens*, and the remaining fractions were divided among archaea and viruses, which combined never exceeded 1% of the classified reads (Fig. 1b, see Table S1 for details).

When we examined only the bacterial taxa present in our datasets, the most abundant phyla across all rivers were Proteobacteria with an average relative abundance of 63.7% (49.1–77.3% per sample), followed by Actinobacteria (20.9% average, 10.8–36.6% per sample), Bacteroidetes (6.6% average, 3.3–13.8% per sample), and Cyanobacteria (4.4% average, 0.3–27.4% per sample) (Fig. S1a). We also examined individual taxa among all bacteria present in our datasets; however, only nine taxa occurred in any sample above 5% relative abundance and of these, only four taxa occurred at this level in more than a single sample (Fig. S1b). To understand the general composition of our river microbial communities we assessed two types of ‘core’ communities present in our samples: the core community that was ‘consistently abundant’ and that which was ‘sporadically abundant’ (Fig. 1c). First, we identified the core community of taxa that were consistently abundant—meaning taxa were both highly abundant (average relative abundance > 0.1%) and simultaneously present frequently (highly abundant in a majority of samples—defined as greater than or equal to 80% of samples). This group consisted of two phyla, Proteobacteria and Actinobacteria, and four classes composed of 58 individual taxa (Fig. 1c). We then asked how core community composition would change if we allowed variation in abundance across samples (i.e., those that are highly abundant in some samples but low in others: sporadically abundant). This flexibility more than doubled the phyla, classes, and number of taxa present and added a key phylum of interest: Cyanobacteria (Fig. 1c).

**Figure 2:**
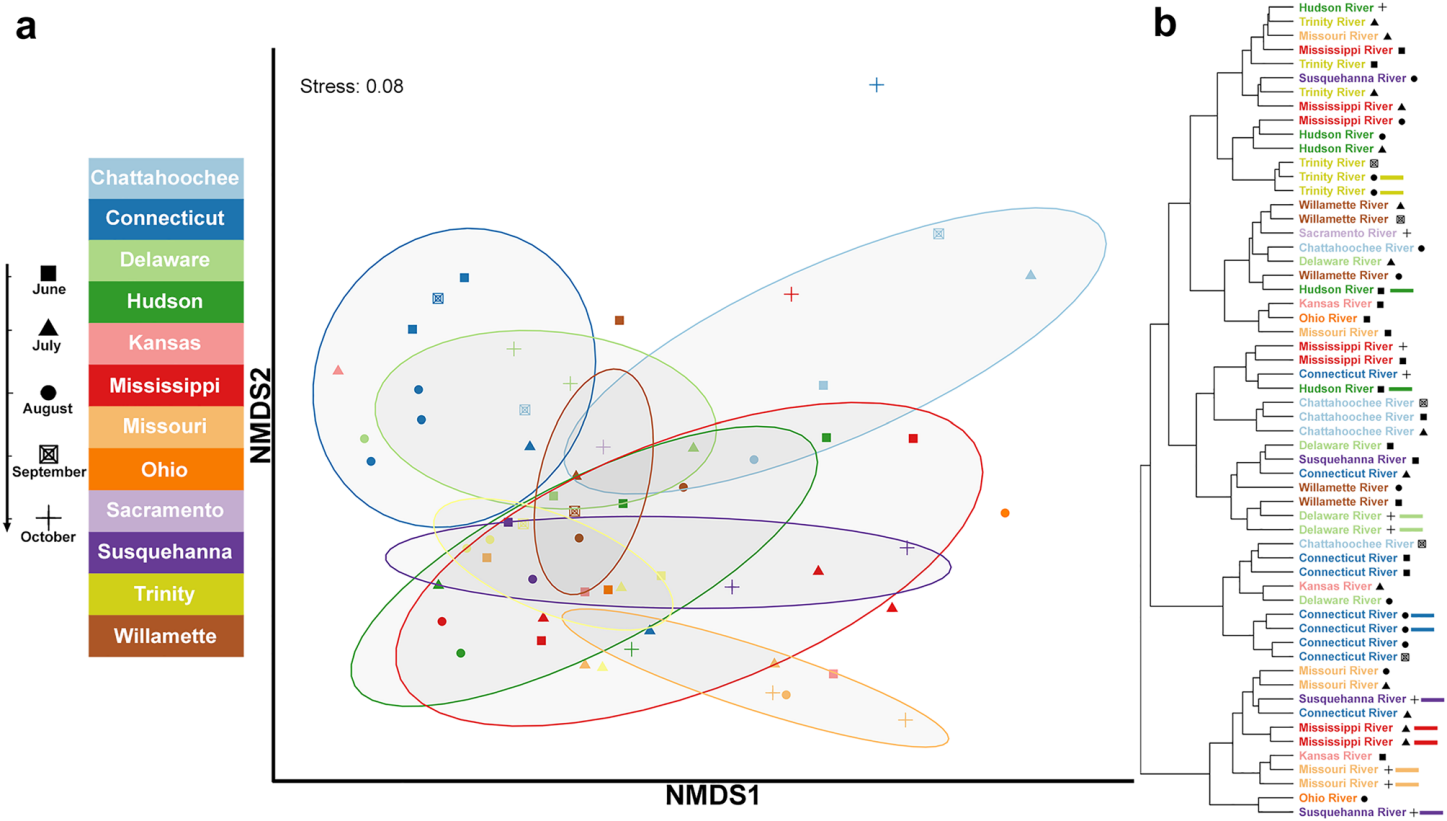
Beta diversity of microbial community composition in U.S. river samples. (**a**) Non-metric multidimensional scaling (NMDS) plot of Bray–Curtis distances for each sample. Points are colored by river, with different shapes representing month (see legend on left). Colored ellipses with confidence levels of 67% were generated for each river with more than two points. (**b**) Cluster diagram of all samples based on Bray–Curtis distance and complete agglomeration clustering algorithm. Month of sample is indicated by shape. Colored bars to the right of samples indicate sequential replicates (replicates are all pairs of samples).

Next, we investigated differences in within-sample diversity among our samples (alpha diversity). We calculated estimates for the Observed Species, Shannon, and Simpson (including Inverse Simpson) diversity indices (Fig. S2a). No rivers had significant differences in the raw number of observed taxa (Kruskal Wallis, *p* = 0.4867). In contrast, Shannon and Simpson diversity indexes were different between rivers (Kruskal–Wallis, *p* = 0.0031 and *p* = 0.0023, respectively), although post-hoc pairwise comparisons via Pairwise Wilcoxon Rank Sum Tests revealed only 4–5 pairwise differences between rivers that were marginally significant (all *p* values were just under 0.05 threshold) (Fig. S2a). To explore possible differences in general microbial community composition between rivers (beta diversity), we performed permutational multivariate analyses of variance (PERMANOVA), clustering analysis, and ordination using non-metric multidimensional scaling (NMDS). Microbial communities of the rivers were found to be significantly different based on both the river identity (Bray–Curtis distances, PERMANOVA; R^2^ = 0.45, *p* < 0.001) and the month during which the sample was collected (R^2^ = 0.12, *p* = 0.02). Further, in a model that considers whether river identity depends/interacts with the date from which they are sampled—we found the variance of the two factors to be ~ 45% and ~ 9%, respectively (based on Bray–Curtis distances, *p* < 0.001). Despite these findings, NMDS (Fig. 2a) and clustering analysis (Fig. 2b) revealed only modest grouping of samples by river, month of sampling, or even by the sequential replicates we included in our analysis (Fig. 2b, colored bars).

### Cyanobacteria and potential cyanotoxin producing species

**Figure 3:**
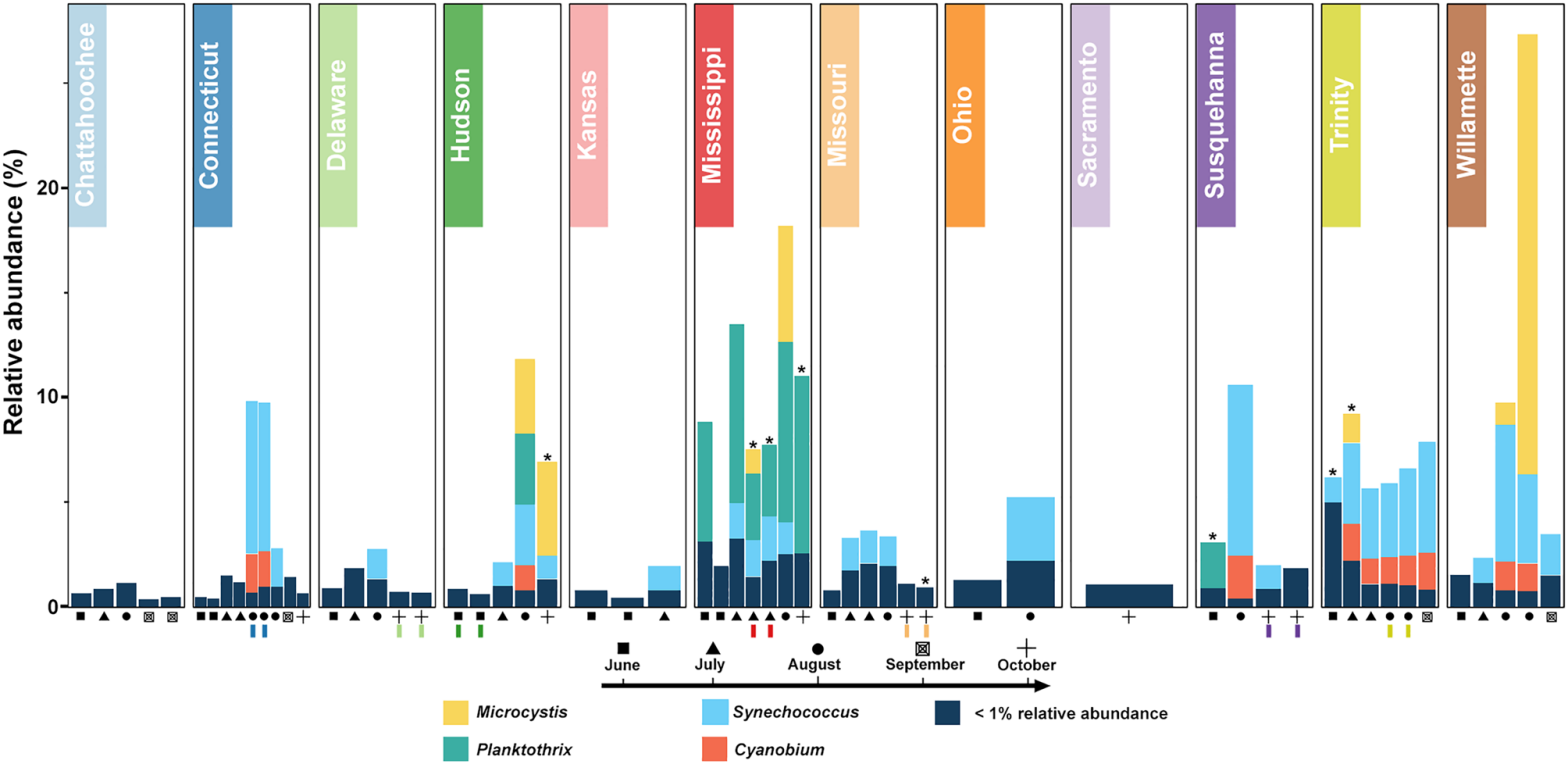
Cyanobacterial genera. Relative abundance of cyanobacterial genera occurring above 1% relative abundance threshold (compared to all bacteria) in each U.S. river at each time point sampled in 2019. Samples are grouped by river (top) and arranged by date (shapes under each bar—see legend below shapes). Samples taken sequentially (replicates) are indicated by colored hashes below date shape. Samples with detectible microcystin toxin levels are indicated by asterisk above bar. Bar color indicates genera as shown by legend.

To begin exploring the cyanobacterial community more closely we first pruned all taxa not belonging to the phylum Cyanobacteria from our dataset. This left 166 taxa among our various river samples (24–153 taxa per sample) comprised of 55 genera (14–55 genera per sample). We then examined the dominant cyanobacterial genera (those with greater than 1% relative abundance compared to all bacteria in any individual sample) (Fig. 3). Only 4 genera were present above 1% relative abundance, highlighting how a significant majority of the cyanobacteria occur at low levels. The highest abundances of any cyanobacteria were in the genera *Microcystis* and *Planktothrix*, which are well-known potential cyanotoxin producers^45^. Furthermore, the relative abundance of *Microcystis* sp. in the Willamette River during August was the second highest relative abundance of any bacteria among all the samples (~ 15% relative abundance to all bacteria, Fig. 3, Fig. S1b). Finally, it is worth noting that genera having < 1% relative abundance include additional potential cyanotoxin producers (e.g., *Dolichospermum*, *Nodularia*, *Cylindrospermum* and *Oscillatoria* among others; see Fig. S3).

To confirm our metagenomics-based findings, a more traditional quantification method (quantitative PCR (qPCR)) was performed. We used assays targeting both the phylum Cyanobacteria as well as the genus *Microcystis* and compared the metagenomics-based quantification of cyanobacteria and *Microcystis* to that of gene copy numbers derived from qPCR standard curves. These data showed significant positive correlation via Pearson correlation analysis (r = 0.6, *p* < 0.001 and r = 0.78, *p* < 0.001, respectively) (Fig. S4a,b), thus adding additional support to our metagenomics-based results.

**Figure 4:**
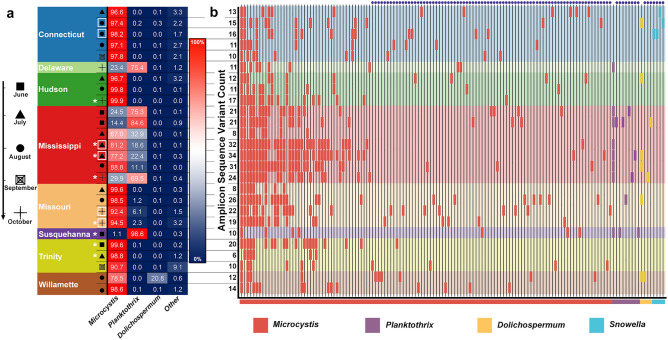
*mcyE* sequencing. (**a**) Percentage of reads from *mcyE* amplicon sequencing that are derived from various potential toxin producing cyanobacterial genera from each U.S. river sample across various sampling months (indicated by shapes, see legend). Greater percentages are shown as dark red, lower percentages as dark blue. Samples that did not possess the *mcyE* sequence (see text) are not included. (**b**) Amplicon sequence variants (ASVs) assigned to each river sample. The legend for river and date of sample in (**a**) applies across the panels to (**b**). Each vertical line represents a unique ASV with bars placed along each line to indicate the presence of that ASV in the corresponding sample row. ASV lines are ordered left to right by abundance and grouped by genus of origin. Total ASV counts per sample are indicated on the left axis line. ASVs unique to a single sample are indicated along the top with purple dots. The color of each bar and the colors along the bottom indicate the genus to which each ASV was taxonomically assigned (legend at bottom). Replicates are indicated by a box around sample date shape. Samples with detectible microcystin toxin levels are indicated by an asterisk.

Next, because potential cyanotoxin producing genera detected by our metagenomics may not necessarily possess the genetic infrastructure required to produce cyanotoxins, we performed amplicon-based next generation sequencing (NGS) to specifically target the *mcyE* gene, a highly conserved gene in the microcystin synthetase gene cluster critical to MC production. Just over half (32) of our samples failed to amplify the *mcyE* gene via PCR indicating that the levels of *mcyE* were below detection limits and thus MC producers were not present at significant levels (Table S2). In the remaining 26 samples (~ 45% of all samples), *mcyE* amplified efficiently, indicating the presence of MC synthetase gene sequences. Among the genera present when the amplicons were taxonomically identified, most *mcyE* sequences originated from the genus *Microcystis* (Fig. 4a,b). In three rivers, however, *Planktothrix-*unique sequences were highly prevalent and, in some cases, the dominant genera of origin for the *mcyE* gene (Delaware, Mississippi, and Susquehanna Rivers (Fig. 4a)). In a single sample from the Willamette River, we also detected ~ 21% of *mcyE* amplicons originating from the genus *Dolichospermum* (Fig. 4a). To understand the genotypic diversity of the *mcyE* gene and the geographic distribution of sequence variation, we then classified the amplified sequences as unique amplicon sequence variants (ASVs)^46^. After some manual curation (see materials and methods) we detected 140 ASVs across our 26 *mcyE* positive samples (Fig. 4b, Fig. S5). The Mississippi River samples possessed the most diverse assembly of ASVs (averaging ~ 24 ASVs per sample) while other locations had fewer (an average of ~ 15 ASVs) (Fig. 4b). A majority of the ASVs (94/140) we detected occurred in single samples (Fig. 4b; dots above each ASV line). We taxonomically classified ASVs (Fig. 4b; bar color and squares along bottom and Fig. S5) and in agreement with our taxonomic distribution analysis (Fig. 4a) determined that a vast majority of ASVs arise from the *Microcystis* genus (123/140; Fig. 4b). Separate and rarer ASVs originated primarily from the genera *Planktothrix* and *Dolichospermum* (Fig. 4b; purple and yellow bars, respectively). One unique set of ASVs arose from the genus *Snowella*, which only occurred in the Connecticut River in July and early August (Fig. 4b, blue bars). Microcystin was detected at low levels (≤ 0.15 µg/L) in eight of the 58 samples included in this analysis^47^. As expected, those samples with detectible MC all contained the *mcyE* gene, although relative abundance of potential MC producers was not a reliable indicator of MC presence/absence (asterisks in Figs. 3, 4 indicate samples with detectible MC levels).

**Figure 5:**
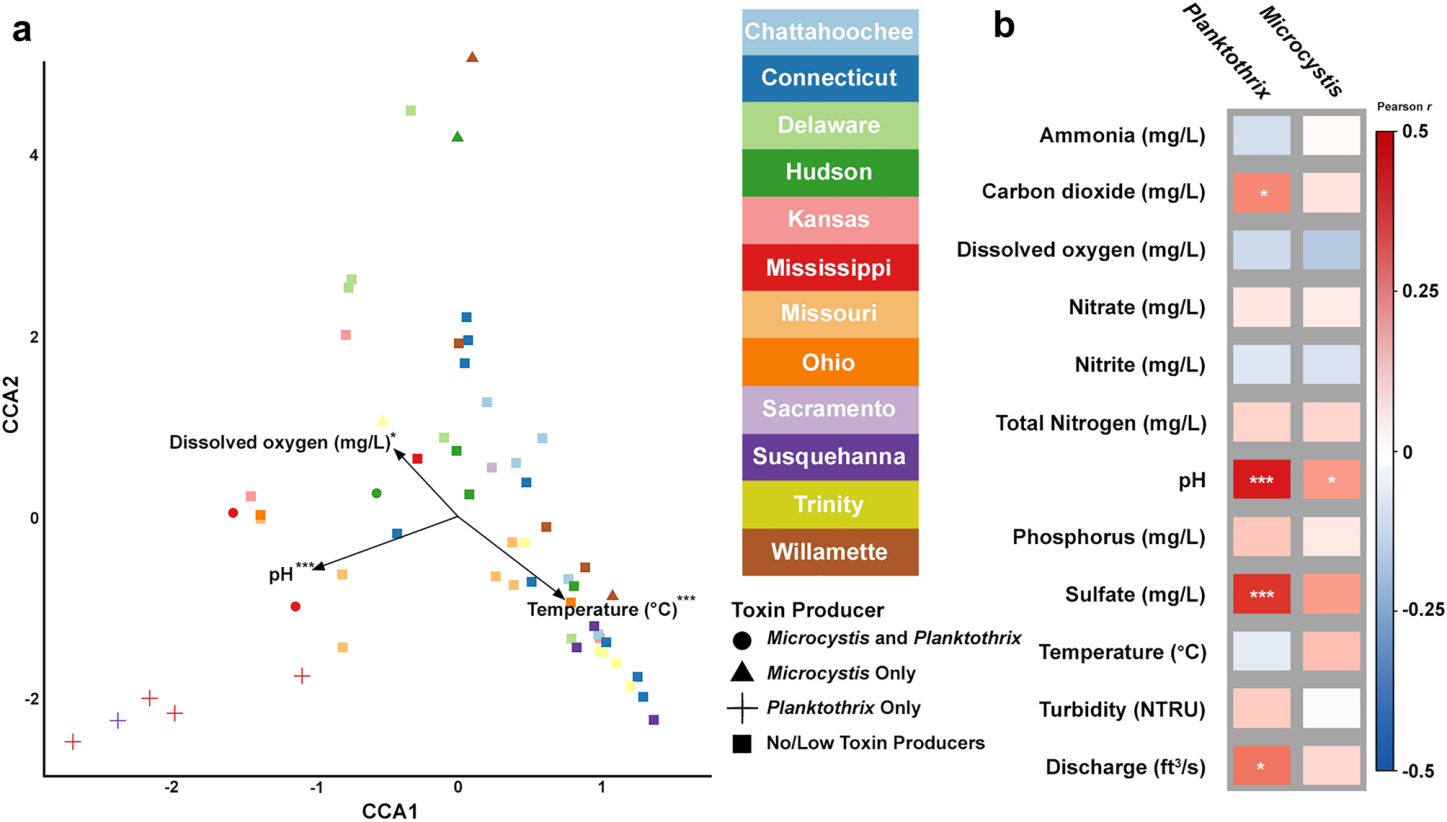
Analysis of cyanobacteria and potential toxin producing species in samples from U.S. rivers and their relationship to environmental variables. (**a**) Canonical Correspondence Analysis (CCA) plot for bacterial species within the phylum Cyanobacteria from each river. Colors indicate river identity. Shape indicates the category of potential cyanotoxin producer contained in each river above 1% relative abundance—either *Microcystis* spp., *Planktothrix* spp., a combination of the two, or no toxin producers (see Fig. 3 for details). The black arrows correspond to the environmental factors that significantly impacted cyanobacterial communities, where the size of each arrow represents degree of significance (longer arrow greater significance). (**b**) Pearson correlation between possible toxin producing genera and environmental variables. The color gradient on the right indicates Pearson correlation coefficients, with more positive values (dark red) indicating stronger positive correlations and more negative values (dark blue) indicating stronger negative correlations. The asterisks denote the significance levels (two-sided) of the Pearson correlation coefficients (n = 58 biologically independent samples). In (**b**) *p* values were adjusted for multiple testing using the Benjamini and Hochberg false discovery rate controlling procedure. For (**a,b**): ***p < 0.001, **p < 0.01 and *p < 0.05.

As the final step in our analysis, we sought to determine how various environmental and physicochemical variables from each river ecosystem may affect the observed differences in abundance of cyanobacterial taxa, as well as the presence/absence of potential cyanotoxin producing genera and/or species. We organized samples (as before—pruned to contain only cyanobacteria) by river identity as well as by the composition of their potential cyanotoxin producers (those occurring above 0.5% relative abundance) and performed Canonical Correspondence Analysis (CCA) (Fig. 5a). CCA showed how the varying cyanobacterial composition of the samples is associated with temperature (*F* = 8.82, *p* < 0.001), dissolved oxygen (*F* = 2.59, *p* = 0.034), and pH (*F* = 11.60, *p* < 0.001) (Fig. 5a). The CCA explained 40% of the variance. None of the remaining environmental variables measured were statistically significant (see Table S3 for all abiotic variables considered). The CCA showed that samples from the Mississippi River containing *Planktothrix* spp. corresponded with increasing pH (red crosses and dots, Fig. 5a), whereas other samples that contained cyanotoxin producers showed less clear correspondence with the significant variables. We did not detect significant correlations with the nutrients nitrogen and phosphorus that are typically associated with varying levels of cyanobacteria^18,48^. We then used Pearson correlations to explore relationships between environmental variables and the abundance of *Planktothrix* and *Microcystis* genera specifically (Fig. 5b). These data further support the observations from our CCA that pH positively correlated with *Planktothrix* abundance (Fig. 5b, r = 0.50, *p*_*adj*_ < 0.001). In addition, our analyses further revealed significant positive correlations between *Planktothrix* and carbon dioxide (r = 0.30, *p*_*adj*_ < 0.04), sulfate (r = 0.48, *p*_*adj*_ < 0.001) and river discharge (r = 0.35, *p*_*adj*_ = 0.02). We also detected a very weak, and marginally significant positive correlation between *Microcystis* spp. and pH (r = 0.26, *p*_*adj*_ = 0.05) (Fig. 5b).

## Discussion

### Overall microbial community of large U.S. rivers

Among the general microbial communities present in the twelve U.S. rivers analyzed in this study, several broad observations can be inferred. First, when sampling DNA from large U.S. rivers the predominant source of DNA is consistently of unknown origin (Fig. 1b). The identity of these sequences was generalized as “unclassified” and while a certain portion are undoubtedly true unclassifiable sequences, a significant portion may be of eukaryotic origin. A large eukaryotic fraction of DNA would match general conclusions drawn from estimating eukaryotic diversity in freshwater ecosystems^49^, but would also conflict with trends from a global long-read based metagenomic study of riverine ecosystems that reported far lower fractions, at less than 5%, of eukaryotic DNA^33^. Without further analysis and customized databases, it is difficult to determine the identity of these DNA sequences. Future work expanding on shotgun and/or long-read metagenomic approaches can be tailored to address the identity and origin of this category of environmental DNA (see^50^ for an example).

The second observation from our metagenomic analysis highlights that, while our samples varied geographically and temporally, the overall microbial communities present in our 58 samples, though not identical, were similar in both taxa diversity and composition. Analysis of beta diversity metrics failed to reveal any single river, region, or time that stood out among the samples (Fig. 2a,b). Within these similar microbial communities overall number of taxa averaged ~ 3000 (Fig. S2, Table S1) matching the richness reported across samples in a global river metagenomic survey in 2020^33^ and similar to reports from metagenomics projects performed on the Mississippi River over the past 10 years^27,29–31,34,35^. When we parsed the ‘core’ communities in our river samples (Fig. 1c), our data also paralleled multiple studies which examined the most consistently abundant taxa in riverine metagenomes^25,27,30–35,51^. These data indicate that microbial communities of large U.S. rivers are generally consistent across geographic location and time, and the differences we detected are a by-product of subtle variation in low abundance taxa. This taxonomic consistency means that samples from the same river at nearly the same time (sequential replicates) can be indistinguishable from samples in a totally different river at a different time. A similar phenomena was also reported in a 2013 analysis of the Mississippi River^27^ and those findings are recapitulated here in the sequential replicates included in our samples (Figs. 2b, 3a). When assessing clustering among the whole microbial community (Fig. 2b), some sequential replicates occur in the same isolated branch of the dendrogram as expected (e.g., Connecticut River: blue bars). In other instances, however, sequential replicates can either be (i) clustered with an entirely different river at an unrelated point in time (e.g., Delaware River replicates in October: light green bars—which are clustered with a Willamette River sample from June); or, (ii) even located in completely different branches with multiple other rivers (e.g., Hudson River replicates: dark green bars). In summary, the taxonomic composition of the river samples is strikingly similar, so much so that even sequential replication fails to consistently define a river’s temporal or geographic identity. An important caveat to these findings is that sampling was limited to the warmest summer months, which may have minimized differences in river microbial communities. This possibility is supported by previous work from a single site in the lower Mississippi River that showed bacterial assemblages change most dramatically along seasonal gradients^30^. Despite these compositional similarities in samples, differences in the abundance of specific taxa were detected, for example, in suites of cyanobacteria.

### Cyanobacteria and cyanotoxin producing taxa of large U.S. rivers

Across our riverine datasets, when we limited our analysis to cyanobacteria, the overall number of taxa averaged just under 100 across our samples (24–153). This taxonomic richness stands in contrast to previous NGS analyses of cyanobacteria done on lentic ecosystems, which often contained far less taxonomic diversity^10,11,15,37,52–54^. We used CCA to assess how total cyanobacterial communities across our sampled rivers may be affected by local environmental variables. Only three of the 11 variables we analyzed were significant (pH, temperature, and dissolved oxygen) partially matching a similar analysis of the Mississippi River which found correlations between pH and cyanobacterial taxa^27^.

One of the main foci of our work centered on examining the cyanotoxin producing taxa present in our river samples. Among all the cyanobacterial taxa we detected were a considerable number of possible cyanotoxin producing species belonging to numerous genera including *Planktothrix, Microcystis*, *Dolichospermum*, *Nodularia*, and others (Fig. S3). While it is possible that any of these taxa could act as seeds for future cyanotoxin producing blooms, most genera occurred at low levels (< 1% relative abundance). In contrast, *Microcystis* and *Planktothrix* spp. were present at much higher levels and their relative abundance varied considerably across both rivers and time. In one sample, the Willamette River in late August, *Microcystis* spp. comprised nearly 15% of the total bacteria present, by far the largest portion of any cyanobacterial taxa. All other river samples tended to have lower, more modest levels of individual taxa. The Mississippi River was unique in that all but one of our seven sampling times contained *Planktothrix* spp. at considerable and consistent relative abundance levels. This was the only highly abundant, potential cyanotoxin producing cyanobacteria across all 58 of our samples to show any bias toward river identity, although we cannot exclude the possibility that this pattern is an artifact of limited sampling in some rivers. We also explored how the environmental variables we assessed via CCA for all cyanobacteria may also correlate with *Microcystis* spp*.* and/or *Planktothrix* spp. directly. Generally, the patterns mirrored our CCA, where pH strongly and positively correlated with *Planktothrix* abundance. Other abiotic factors did correlate significantly, albeit marginally so, with the abundance of the two genera, although most factors showed no correlation. These data imply that for large U.S. rivers, most measured abiotic factors have modest relations with overall cyanobacteria abundance and an indirect influence on *Microcystis* and *Planktothrix* spp. For lentic aquatic ecosystems, nutrient concentrations (nitrogen, phosphorus, etc.) and abiotic measures are often used to predict trophic level and the likelihood of rapid expansions in cyanobacterial populations^18,48^. We also examined the trophic status of the rivers included in our study. Samples from a majority of the rivers qualified as eutrophic or above by our estimates (Table S4 and see Ref.^55^), yet these rivers did not present dramatically different cyanobacterial community composition or richness compared to other sampled locations. All together, these findings contrast with existing literature from lentic ecosystems where environmental factors are closely intertwined with cyanobacterial communities^56–58^. Instead, the dynamic, multi-source, lotic environment of a river seems to minimize the influence fluctuations in abiotic environmental variables can impart on the cyanobacteria present in the river at any given time, and any impact these variables may have is quickly overwhelmed by the tumultuous nature of the riverine ecosystem. One possibility to explain this conflicting finding is the scale of our study. Samples were collected monthly to bi-weekly; however, changes at the bacterial level are likely occurring much faster and we thus failed to capture those processes. Another possibility is the lens through which we focused our analysis. Namely, the act of placing cyanobacterial abundance within the framework of the entire bacterial community and separate from the phytoplankton community and overall algal biomass. When examined in this context it appears that the bulk of a river’s microbial community is likely determined by smaller scale upstream inputs within the watershed, where the origin of specific taxa may be from relatively distant and separate ecosystems such as upstream lakes and ponds—a phenomenon suggested previously for large rivers^18–21^. In the case of certain environmental factors (e.g., pH), cyanobacteria can also be causative rather than reactive^59,60^. This, in turn, may mean that rivers such as the Mississippi, which had the most substantial levels of *Planktothrix* spp. and the highest pH, may present these two correlated factors because of an active *Planktothrix* bloom (either at the sampled location or points upstream) with concomitantly high pH. Parsing the specific origin of individual taxa within riverine ecosystems and tracing them to adjacent water bodies is challenging. Future studies could explore these questions by implementing additional genotypic information. As a first step toward such an analysis we also explored sequence distribution and variation within the *mcyE* gene region.

### mcyE sequencing and the distribution and genotypes of potential cyanotoxin producing taxa

One key caveat to metagenomics-based analyses of cyanotoxin producing cyanobacteria is that taxa which lack the genetic infrastructure necessary to produce cyanotoxins cannot be differentiated from toxin-producing taxa. This caveat can obfuscate interpretations made from raw presence/absence data. To address this possibility and interrogate the cyanotoxin producing potential of large U.S. rivers, we performed deep sequencing on amplicons derived from the *mcyE* gene; a critical component of the polycistronic gene region necessary for synthesis of one of the most commonly occurring cyanotoxins worldwide: MC^44^. Along with confirming the cyanotoxin producing potential of various taxa, these data can also be used to explore the genotypic distribution and diversity of the *mcyE* gene—represented as ASVs, a modern replacement for operational taxonomic units (OTUs)^46^. Our analysis revealed that most river samples did not contain *mcyE* (or at least it could not be uniquely amplified by PCR), and taxa therein cannot, at the given sample point, produce MC. Twenty-six river samples (~ 45%) did contain taxa possessing the *mcyE* gene, and in most cases those samples with the highest metagenomics-based relative abundance of potential cyanotoxin producers were among these samples. These findings are generally in agreement with previous qPCR-based analyses done on the same rivers in previous years^38,39^. This finding indicates that U.S. rivers that contain the highest levels of known cyanotoxin producing taxa often also contain genetic indicators for cyanotoxin production (again, at least for MC). One important caveat to these findings is that only eight of our samples contained detectible concentrations of MC at the time of sampling^47^ and those samples with detectible MC did not necessarily have the highest relative abundance of potential cyanotoxin producing cyanobacteria. Nevertheless, our *mcyE* sequencing showed considerable genotypic diversity (ASVs) among our samples; a majority of which was distributed primarily among the *Microcystis* genus (123/160 ASVs). *Planktothrix* was the second most common origin of the *mcyE* sequences in our samples, distributed across three of the rivers and, at times, contributing a majority of the *mcyE* sequences we detected (Delaware, Mississippi, and Susquehanna Rivers; Fig. 4a). Further, it appeared that a single *Planktothrix*-derived *mcyE* variant was the dominant contributor to these three rivers, as only a single ASV was consistent across them (Fig. 4b). Additionally, three ASVs derived from the genus *Snowella* were detected only in the Connecticut River—representing the only single-river-unique, *mcyE* possessing genera (Fig. 4b). Unfortunately, this genus was not detected in our metagenomic analysis, even among low abundance cyanotoxin producers (Fig. S3) so we cannot determine the relative abundance of taxa belonging to this genus nor can we rule out the possibility that this is an artifact of our *mcyE* sequencing and/or a misclassification of the ASV (although we confirmed the classification via multiple methods and see Fig. S5).

In summary, these data further accentuate the high levels of taxonomic and now genotypic diversity present in large U.S. rivers. The level of diversity is especially pronounced when we examine previous analyses performed with technically similar *mcyE*-sequencing approaches, but performed on lentic ecosystems with ongoing cyanobacterial blooms, which reported dramatically fewer sequence variants (2–19 variants)^10,15^. Ultimately, future studies could explore how ASVs can be used to trace specific variants across space and time^21^, and/or be used to investigate how particular MC congeners (variant forms of MC) are associated with specific sequence variants and how they are distributed across ecosystems.

### Conclusions and future directions

Cyanobacteria and associated cyanotoxins represent a considerable challenge that can impact public health, recreation, and economies. Understanding the complex suite of variables that precede, accompany, and are produced by blooms of cyanotoxin producing cyanobacteria requires a holistic approach, mandating studies focused on both bloom events, as well as pre- and post-bloom time periods; and, across these periods, also performed on the full spectrum of aquatic freshwater ecosystems—from small lentic ponds to large lotic riverine ecosystems. Here we used NGS and metagenomics to better understand the microbial communities, cyanobacteria, and cyanotoxin producing taxa present in twelve large U.S. rivers during ambient non-bloom conditions across the summer months of 2019. Through our analysis we revealed that large U.S. rivers are taxonomically rich, but overall consistent within these communities across spatial and temporal metrics. Nevertheless, subtleties in relative abundance of some taxa, such as possible cyanotoxin producing cyanobacteria, drive overall significant differences between rivers and time. These cyanobacteria are present at considerable levels in some rivers, and with the confirmed presence of the genetic loci necessary for production of cyanotoxins could, if conditions become favorable, act as seeds for the development of cyanotoxin producing blooms. This study failed to find an abundance of significant correlations between abiotic environmental variables and cyanobacteria and/or potential cyanotoxin producing taxa. This finding is likely a product of scale. Large riverine ecosystems are a challenge to characterize with relatively small individual snapshots of the dynamic ecosystem. Future studies should be especially cognizant of these challenges and updated sampling strategies may mitigate some of these effects and lead to a clearer perspective on environmental-microbial relationships (for example see^50,61^). Additionally, this work is among the first to provide data on the genotypic diversity of the *mcyE* gene and reveals the immense sequence variation that exists in large riverine ecosystems compared to lentic bloom-focused events and locations. Ultimately, these data can be used to trace the flow of specific sequence variants across and through ecosystems and can be a powerful future tool to understand the origin and evolutionary history of variants driving bloom events. In conclusion, our work provides a first look at microbial communities and potential cyanotoxin producing cyanobacteria in large U.S. rivers using NGS approaches and can be used to better understand the taxonomic and genetic conditions that exist within ambient, non-bloom conditions in lotic riverine ecosystems.

## Materials and methods

### Study sites

All 12 study sites are large inland or coastal rivers routinely sampled as part of the USGS National Water Quality Network and equipped with streamflow gaging stations^62^. These sites were selected to include a diverse range of drainage areas, streamflow conditions, and trophic states throughout the U.S. (Fig. 1a, Table S4).

### Sample collection and DNA extraction

All rivers were sampled during June–October 2019. Samples were collected via near-surface grabs at the centroid of flow in autoclaved and bleached 500 mL polypropylene bottles. Sequential replicates of multiple sample points were taken by immediately repeating the surface grab in a separate bottle. The sample was filtered through a 0.4 µm polycarbonate filter and filters were collected in individual tubes for DNA extraction. For DNA extraction, 400 µL of 1× Tissue & Cell Lysis Solution (Epicentre Technologies Corp., Madison, Wisconsin, USA), was added to each tube. Each tube was bead-beaten once for 1 min using a Mini-Beadbeater-16 (BioSpec Products, Inc., Bartlesville, Oklahoma, USA). Tubes were then centrifuged at 12,000×*g* for 5 min at room temperature and the supernatant was transferred to a sterile microcentrifuge tube. One (1) μL of 50 μg/μL Proteinase K was added to each sample and incubated for 15 min at 65 °C. After incubation, 1 μL of Rnase A was added to each sample and then they were incubated for 30 min at 37 °C. Two hundred (200) µL of MPC protein was then added to each sample. They were then put on ice for 5 min before being centrifuged at 15,000×*g* for 10 min. DNA purification was then performed by using a ZymoBIOMICs DNA Miniprep Kit (Zymo Research). Once completed, samples were stored in a − 20 °C freezer for future downstream analyses. DNA concentration was quantified using a Qubit™ machine.

### Illumina MiSeq metagenomic sequencing and read processing

For sequencing, libraries were prepared using a Nextera XT DNA Library Prep kit following manufacturer’s protocol. The libraries were denatured and diluted using a MiSeq Denature and Dilute library guide. The pooled library was then loaded into a MiSeq Reagent Kit v3 (https://support.illumina.com) and run using 300 bp paired-end chemistry (Illumina, San Diego, CA, USA). Our sequencing effort produced an average of 1.9 million paired reads per sample. See Table S1 for additional sequencing details. Raw reads with primers and adapters removed were then processed. Reads were quality checked using Fastqc v.0.11.9^63^ and cleaned using Trimmomatic v.0.39^64^ to perform quality trimming. Trimmomatic was run with the following parameters, LEADING:10 TRAILING:10 SLIDINGWINDOW:5:20 MINLEN:50. After trimming, Fastqc was run again. For all samples an average of ~ 7% of reads were removed by trimming (~ 4% to ~ 17%). The trimmed reads were then taxonomically identified using Kraken2 v.2.1.1^65^ with the Kraken2 standard database using default settings. After identification, the kraken report files were further analyzed using Bracken v2.6.1^66^ to compute abundance of species using default settings for the appropriate read length. Lastly, we generated a single biom formatted output file (specifically JavaScript Object Notation, json format) using the *kraken-biom* function. This file was then used for downstream analyses.

### mcyE PCR, library preparation, and sequencing

Primers were designed by using gene specific sequences for the cyanobacteria *mcyE* gene (Table S5)^44^. Adaptor sequence addition, library preparation and sequencing were performed as described in Ref.^67^ with the following modifications. The first round of PCR was done with 17 µL Accuprime pfx supermix (Thermofisher), 0.5 µL of each primer at 10 µM concentration, and 2 µL of DNA. Positive (DNA isolated from lab cultured water with known *mcyE* containing taxa including *Planktothrix* sp. and *Microcystis* sp.) and negative (blank water) controls were included. Samples that failed to amplify, as determined by gel electrophoresis, were removed from further analyses. After gel confirmation of amplification products, PCR products were cleaned with 14 µL of AMPure XP beads (Beckman Coulter) on 17 µL of PCR product and eluted in 40 µL 10 mM Tris pH 8.5. PCR products were normalized to 20 ng/µL and index PCR was performed using Accuprime pfx supermix, cleaned using 19 µL AMPure XP to 17 µL of PCR product, and eluted with 27 µL of 10 mM Tris pH 8.5. Libraries were normalized to a concentration of 2 nM and 5 µL of each were pooled for a single run of sequencing using a 2 × 300, 600 cycle V3 MiSeq sequencing kit according to manufacturer’s protocol.

Sequencing efforts produced an average of ~ 175,000 paired reads per sample. See Table S2 for additional sequencing details. After removing primers and adaptors, reads were merged using FLASH v1.2.11^68^. For all but seven samples, greater than ~ 95% of reads successfully merged to form single long reads. The seven samples mentioned above had extremely poor combination rates (as low as 45%), and we thus assumed that our first round PCR (above) incorrectly amplified spurious sequences rather than the *mcyE* gene—again, likely because it was not present within the sample. Accordingly, these seven samples (along with the 25 samples that failed to amplify in the first round *mcyE* PCR—see above) were dropped from further analysis. In summary, 26 of our 58 samples were analyzed (see Table S2 for additional details). The merged reads were taxonomically identified using Kraken2 v.2.1.1^65^ with a custom database. Kraken2 report files were then parsed to quantify the proportion (percent) of *mcyE* amplicons arising from possible cyanotoxin producing species. To understand the genotypic diversity present among our 26 samples and within the *mcyE* gene region we used QIIME2 v2021.4.0^69^ to generate amplicon sequence variants (ASVs) with dada2 v1.18.0^70^. We translated each ASV and aligned them using MUSCLE in MEGAX version X^71^ to manually identify sequences that did not code for the McyE protein or those sequences that contained stop codons. These ASVs were removed from the analysis. We assigned each ASV that passed these quality checks to the respective river samples. We used Kraken2 custom databases and BLAST v2.9.0 to assess the taxonomic origin of each ASV. Finally, we generated a phylogenetic tree. Translated protein alignments were generated and manually inspected and curated where appropriate. The evolutionary history was inferred using the Neighbor-Joining method. The bootstrap consensus tree was inferred from 500 replicates. The percentage of replicate trees in which the associated taxa clustered together in the bootstrap test are shown next to the branches. The evolutionary distances were computed using the Poisson correction method and are in the units of the number of amino acid substitutions per site. This analysis involved 153 amino acid sequences. All positions containing gaps and missing data were eliminated (complete deletion option).

### qPCR analysis of cyanobacteria and *Microcystis* spp.

*Microcystis* and Cyanobacteria targets were identified and measured using quantitative polymerase chain reaction (qPCR). Power SYBR PCR Kit (Thermo Fisher), was used to quantify these targets on a QuantStudio 5 and 6 System (Thermo Fisher Scientific, Waltham, WA). PCR reactions were set up by adding 2 μL of DNA to the following master mix: 10 μL of 2× SYBR Green, 5 μL of nuclease-free H2O, 2 µL of 1 ng/μL Bovine Serum Albumin fraction V, 0.5 μL of a 10 μM forward primer, and 0.5 μL of a 10 μM reverse primer. The primers used for Cyanobacteria 16S rRNA were CYAN108F and CYAN377R^72^ while the primers used for 16s rRNA in *Microcystis* were MIC209F and MIC409R^73^. The following thermal programs were applied following default conditions: 95 °C for 10 min, followed by 40 cycles of 95 °C for 15 s. The annealing temperature varied between the two targets, cyanobacteria 16s rRNA primers were annealed at 56 °C for 30 s followed by an extension step at 72 °C for 30 s and *Microcystis* specific primers were annealed at 64 °C for 1 min. A melting curve stage analysis was done for 15 s at 95 °C with dissociation from 60 to 95 °C. Each sample was run as a series containing one undiluted sample, followed by a tenfold dilution to check for inhibition. A no-template negative control lacking spiked DNA was also included. For each target, tenfold dilutions series were generated from corresponding plasmids of varying concentrations. Undiluted plasmid concentrations for Cyanobacteria were 4.78 × 10^7^ ng/μL while *Microcystis* was 6.42 × 10^7^ ng/μL respectively. See Table S5 for primer details. Final gene copy numbers per sample were adjusted for initial raw filtration volume of collected sample.

### Data analysis

Analysis of the final dataset was performed in R v3.5.3^74^ using the packages phyloseq^75^, vegan^76^, and ggplot2^77^. Various alpha diversity estimates (Chao1, Shannon Index, Simpson Index, InvSimpson Index) and between-Sample distances (Bray–Curtis) were computed. Distance matrices were then used to cluster samples using non-metric multidimensional scaling (NMDS). Kruskal–Wallis and post-hoc Pairwise Wilcoxon Rank Sum Tests were used to test for differences in alpha diversity. PERMANOVAs using the *adonis* function on the Bray–Curtis distance matrix with 999 permutations were used to test for statistically significant differences in microbiota composition and diversity between sample groups. Prior to these analyses, data were also checked for overdispersion using the *betadisper* function in the Vegan package^76^. ANOVAs were used to test for the impact of environmental variables on Cyanobacteria in our CCA plot. To both (i) compare environmental variables to fluctuations in potential cyanotoxin producing species and (ii) understand the relationship between metagenomics and qPCR-derived quantification of cyanobacteria and *Microcystis* species, we calculated correlation coefficients for the various datasets. When multiple comparisons were made, *p* values were adjusted for multiple testing using the Benjamini and Hochberg false discovery rate controlling procedure. Where appropriate, data were log transformed and for all tests assumptions of normality and homoscedasticity were validated visually (with Q–Q plots) and statistically (using Levene’s test for equality of variance) to determine appropriate tests.

## Supplementary Information

Supplementary Information.

## Data Availability

All data have been deposited in the NCBI sequence read archive at Accession Number: PRJNA765194. Water-quality data are available through the USGS National Water Information System^78^. Microcystin data are available in Graham et al.^47^.
