# Supplementary material for: Metagenomic mapping of cyanobacteria and potential cyanotoxin producing taxa in large rivers of the United States
Source: Sci Rep. 2023 Feb 16;13:2806. doi: 10.1038/s41598-023-29037-6 (PMC9935515; doi:10.1038/s41598-023-29037-6)
Supplement: Supplementary file 1 — Supplementary Information. [file 41598_2023_29037_MOESM1_ESM.pdf]

SUPPLEMENTARY FIGURES AND TABLES

Supplementary Figures

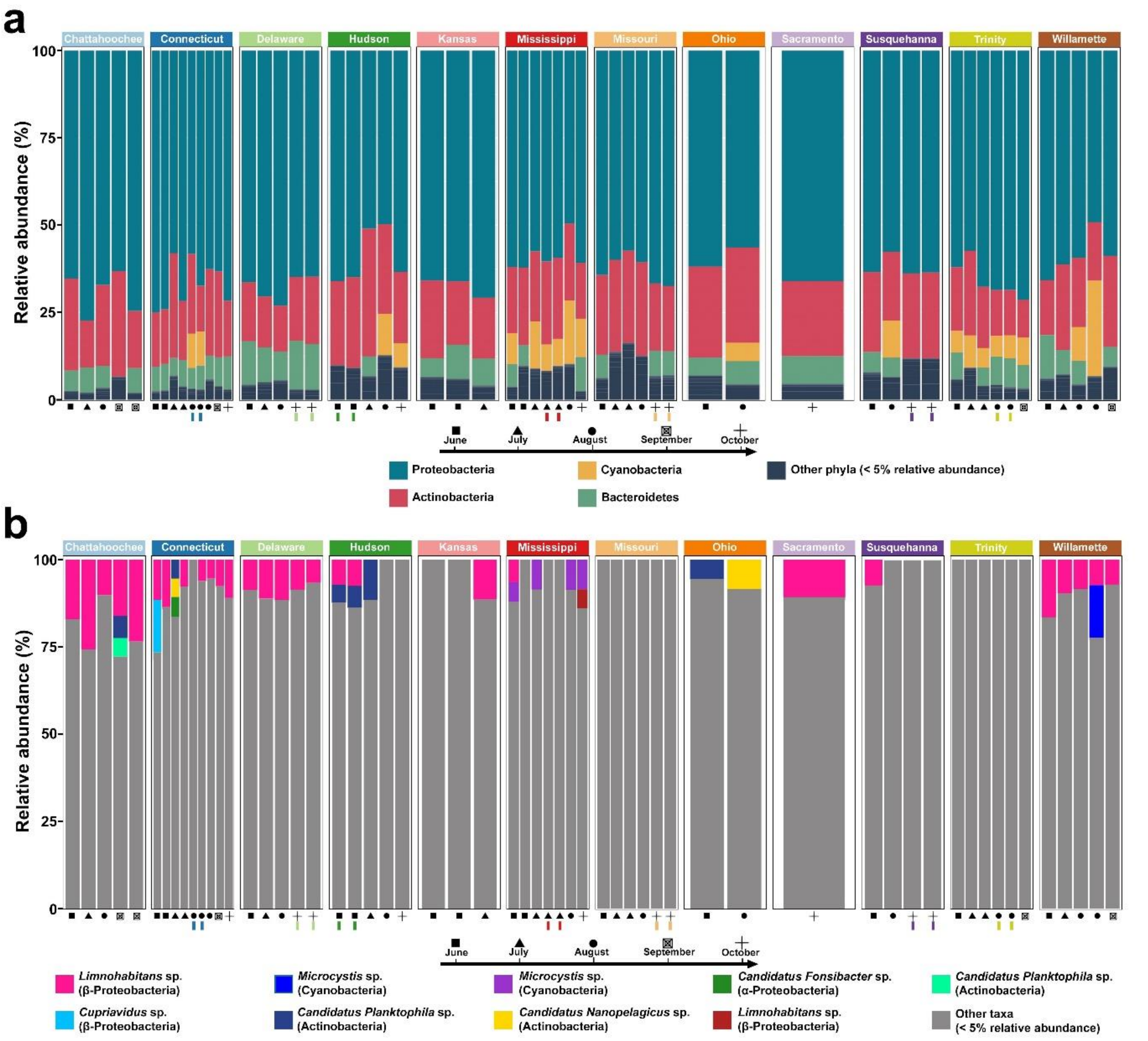

Supplementary Figure S1. Relative abundance of bacterial phyla and taxa occuring above 5% (compared to all bacterial species) in each U.S. river at each time point sampled. (a) Taxa grouped at the phylum level. (b) Individual taxa with respective phylum or class (classes of Proteobacteria) shown in parenthesis. Samples taken sequentially (replicates) are indicated by colored hashes below date shape.

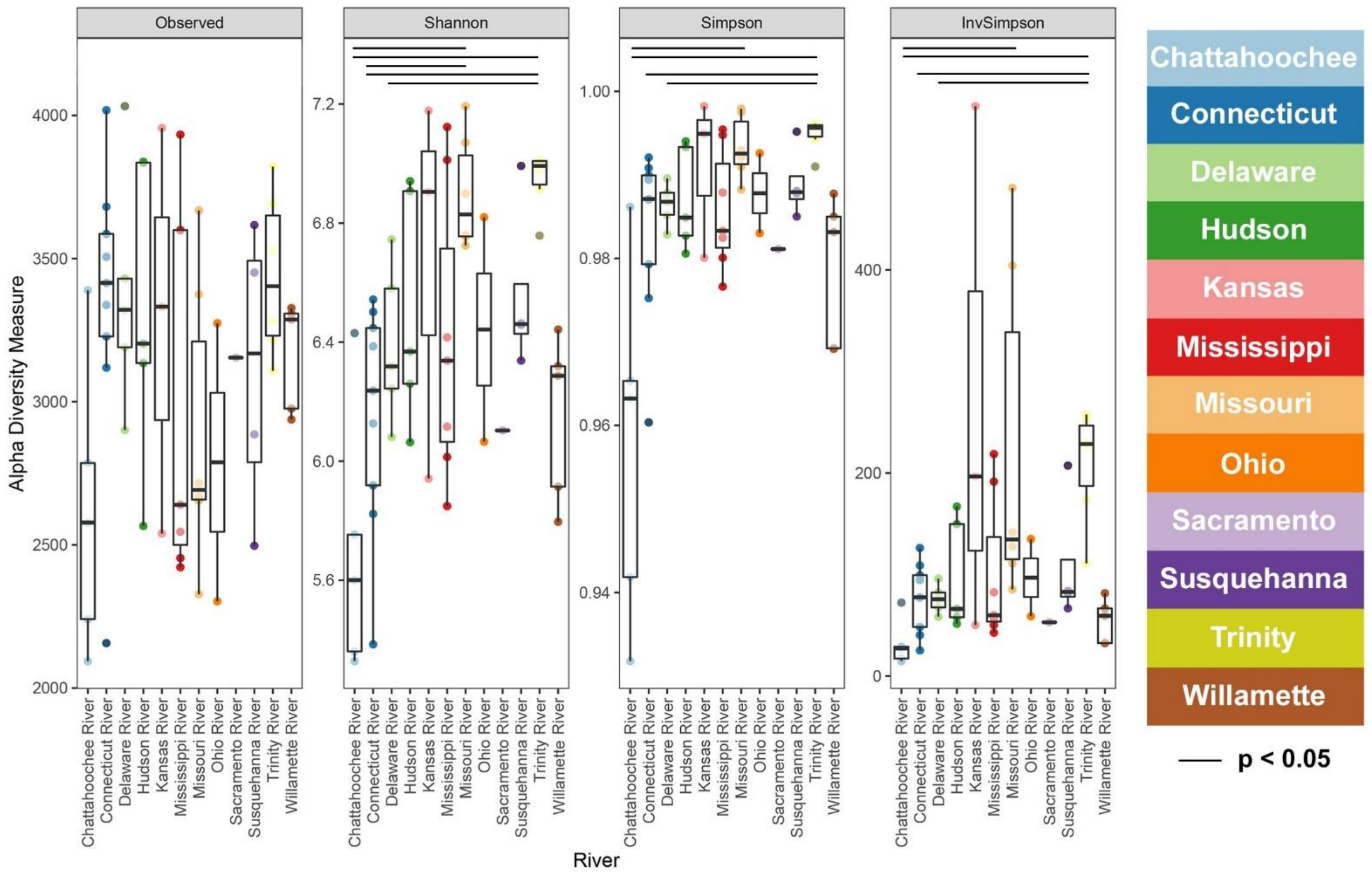

Supplementary Figure S2. Alpha diversity of within-sample microbial community composition in samples from U.S. rivers. Alpha diversity measures for each river sampled including observed (raw) taxonomic diversity, Shannon, Simpson, and Inverted Simpson indexes. Data are shown as a boxplot with individual data points included. Lines between rivers across the top represent pairwise differences below p = 0.05 threshold (Pairwise Wilcoxon Rank Sum Test).

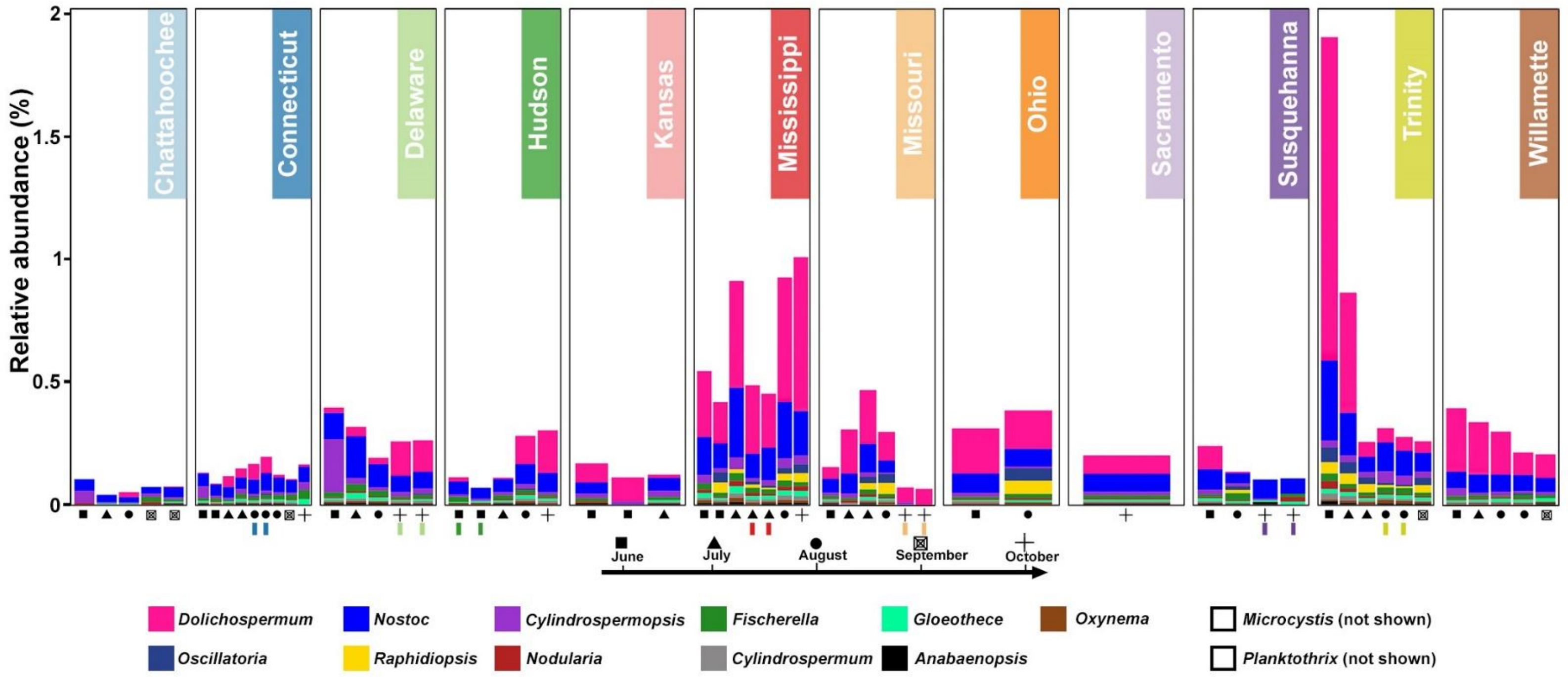

Supplementary Figure S3. Relative abundance of low abundance potential toxin producing bacteria genera in each U.S. river at each time point sampled (compared to all bacterial tax). Taxa grouped at the genera level. Taxa belonging to the *Microcystis* and *Planktothrix* genera are not shown despite being highly abundant potential toxin producers (see Fig. 3) as their high abundance occluded visualization of the lower abundance genera. Samples taken sequentially (replicates) are indicated by colored hashes below date shape.

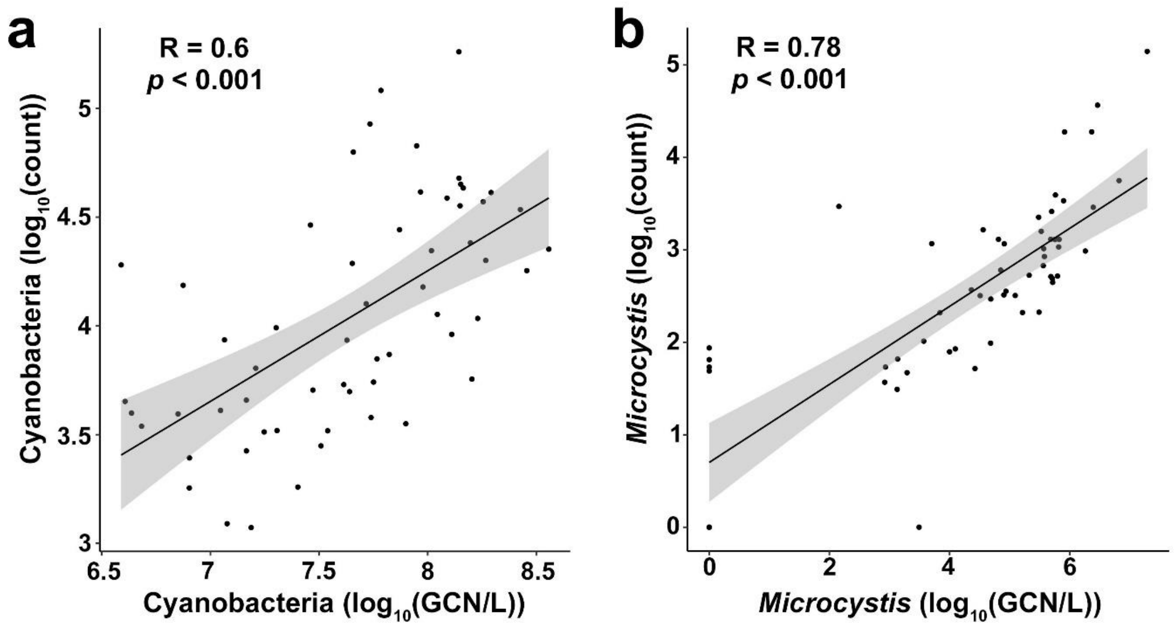

Supplementary Figure S4. Pearson correlation coefficient (R) and P-value (p) between quantitative PCR (qPCR) and metagenomics quantification methods (log scale). (a) Quantification of cyanobacteria. (b) Quantification of *Microcystis* species. Count = raw Bracken-derived abundance count of phylum (a) or genus (b) of interest. GCN/L = gene copy number per liter of sample water.

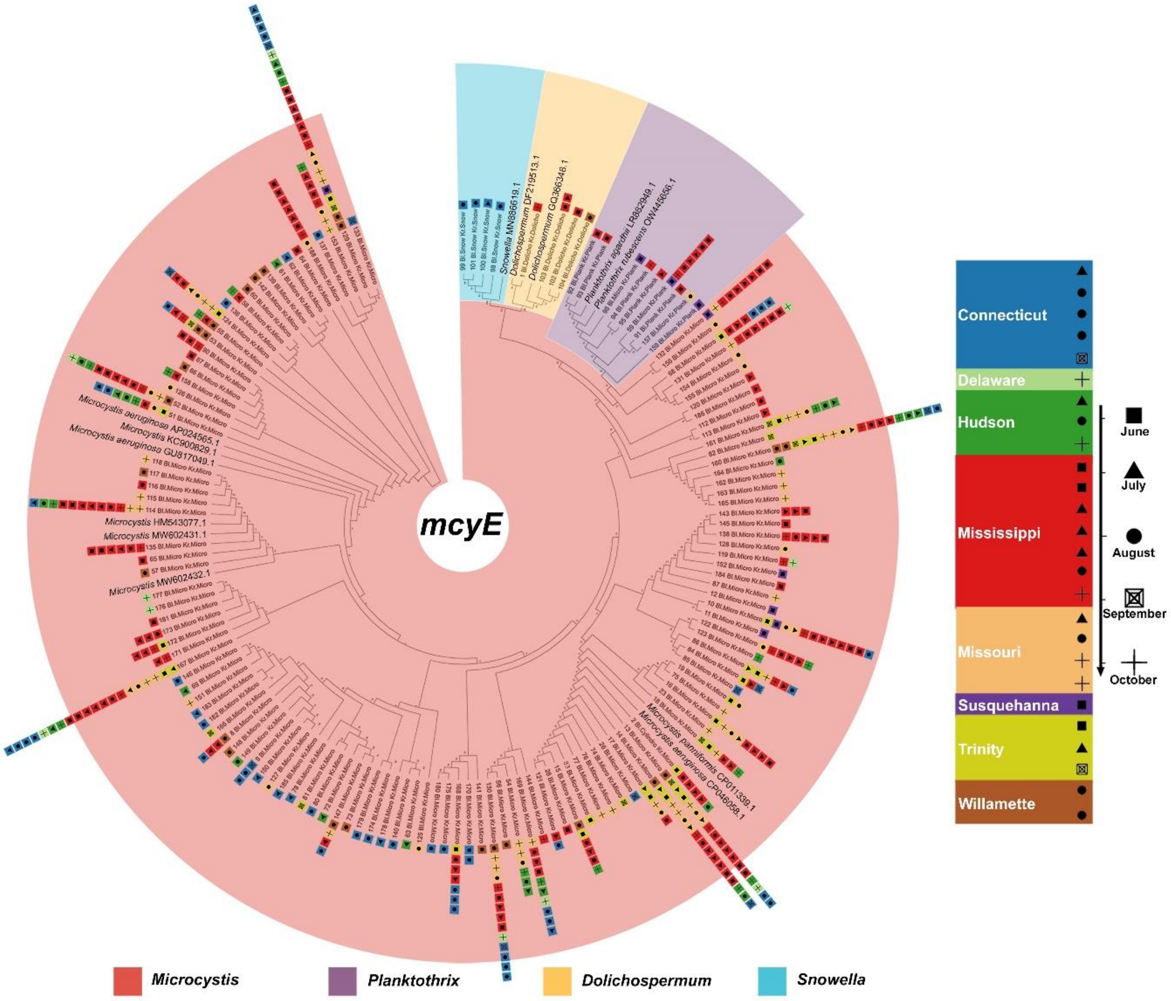

Supplementary Figure S5. Cyanobacteria *mcyE* phylogeny. A neighbor-joining phylogenetic tree based on an amino acid alignment of 140 *mcyE* amplicon sequence variants (ASVs) from 26 U.S. rivers positive for *mcyE* detection and a selection of *mcyE* sequences from known taxa (accession is indicated next to label). The bootstrap consensus tree was inferred from 500 replicates. Color surrounding tree indicates clades belonging to each genus as shown in bottom legend. Each ASV label contains a unique numerical identifier and includes the taxonomic classification inferred from both BLAST (Bl) and Kraken2 (Kr). The squares along each ASV label indicate river identity (color of square – see right legend) and date (shape within square – see right legend) where a given ASV was detected. Micro is *Microcystis*, Plank is *Planktothrix*, Dolicho is *Dolichospermum*, and Snow is *Snowella*.

Supplementary Tables

Supplementary Table S1. Sample and sequencing information for microbial communities in U.S. rivers in 2019. One eukaryote was purposely included: *Homo sapiens*.

Sample ID	River	Location	Date of Sample	Pairs of Reads	Total Bases (bp)	Pairs Surviving Trimming (%)	Reads Unclassified by Kraken2 (%)	Reads Classified by Kraken 2 (%)	Distribution of Classified Reads (%)					Total taxa	Bacterial taxa
									Homo sapiens	Bacteria	Viruses	Archaea	Unkown		
4996-01	Chattahoochee River	Whitesburg, GA	6/5/2019	1,143,427	6.86E+08	94.8	59.2	40.8	1.2	98.4	0.2	0.5	0.5	2578	2573
5046-01	Chattahoochee River	Whitesburg, GA	7/2/2019	910,276	5.46E+08	95.0	57.9	42.1	0.9	98.7	0.2	0.5	0.3	2094	2090
5197-01	Chattahoochee River	Whitesburg, GA	8/19/2019	1,146,928	6.88E+08	95.0	61.2	38.8	0.7	98.8	0.1	0.4	0.2	2786	2783
5260-01	Chattahoochee River	Whitesburg, GA	9/11/2019	1,082,183	6.49E+08	96.1	60.5	39.5	1.1	98.5	0.1	0.3	0.2	2241	2240
5300-01	Chattahoochee River	Whitesburg, GA	9/30/2019	1,941,958	1.17E+09	95.4	57.1	42.9	0.6	99.1	0.1	0.3	0.3	3389	3382
5008-01	Connecticut River	Thompsonville, CT	6/12/2019	2,899,570	1.74E+09	92.5	67.4	32.6	2.5	97	0.1	0.2	0.3	3506	3501
5038-01	Connecticut River	Thompsonville, CT	6/26/2019	2,002,564	1.20E+09	94.7	64.9	35.2	1.9	97.6	0.2	0.4	0.3	3228	3222
5058-01	Connecticut River	Thompsonville, CT	7/10/2019	1,689,644	1.01E+09	91.1	73.6	26.4	3.6	95.1	0.5	0.5	0.5	3119	3061
5104-01	Connecticut River	Thompsonville, CT	7/24/2019	2,074,000	1.24E+09	92.4	69.6	30.5	1.6	97.8	0.2	0.4	0.4	3415	3403
5155-01	Connecticut River	Thompsonville, CT	8/7/2019	2,975,538	1.79E+09	91.5	66.9	33.1	1.9	97.6	0.1	0.3	0.2	4018	3996
5155-02	Connecticut River	Thompsonville, CT	8/7/2019	2,349,730	1.41E+09	92.9	66.9	33.1	1.7	97.7	0.4	0.6	0.4	3681	3663
5218-01	Connecticut River	Thompsonville, CT	8/21/2019	2,159,357	1.30E+09	93.3	64.0	36.0	0.7	99	0.1	0.3	0.4	3586	3579
5244-01	Connecticut River	Thompsonville, CT	9/4/2019	2,415,104	1.45E+09	88.9	69.8	30.2	1.4	98.2	0.1	0.3	0.2	3338	3333
5314-01	Connecticut River	Thompsonville, CT	10/9/2019	1,289,478	7.74E+08	88.9	71.6	28.4	7.5	91.8	0.1	0.6	0.4	2157	2151
5025-01	Delaware River	Trenton, NJ	6/20/2019	1,928,109	1.16E+09	93.1	67.0	33.1	1.9	97.5	0.1	0.2	0.2	3430	3400
5051-01	Delaware River	Trenton, NJ	7/9/2019	1,354,230	8.13E+08	91.6	66.6	33.4	2.0	97.3	0.1	0.2	0.1	2901	2894
5142-01	Delaware River	Trenton, NJ	8/5/2019	3,282,687	1.97E+09	93.7	66.2	33.8	1.4	98.3	0.1	0.3	0.3	4032	4013
5321-01	Delaware River	Trenton, NJ	10/21/2019	2,353,480	1.41E+09	87.9	70.8	29.2	2.4	97	0.4	0.5	0.4	3190	3183
5321-02	Delaware River	Trenton, NJ	10/21/2019	2,536,209	1.52E+09	89.5	68.5	31.5	2.4	97.2	0.1	0.3	0.3	3321	3313
5030-01	Hudson River	Poughkeepsie, NY	6/25/2019	1,817,712	1.09E+09	95.0	71.6	28.4	6.8	92.4	0.3	0.6	0.4	3135	3121
5030-02	Hudson River	Poughkeepsie, NY	6/25/2019	1,265,391	7.59E+08	94.8	70.2	29.8	6.7	92.6	0.4	0.6	0.4	2566	2560
5117-01	Hudson River	Poughkeepsie, NY	7/24/2019	2,430,930	1.46E+09	93.2	71.1	28.9	4.2	94.8	0.1	0.1	0.2	3838	3773
5226-01	Hudson River	Poughkeepsie, NY	8/26/2019	2,365,949	1.42E+09	93.3	73.5	26.5	4.8	94.1	0.2	0.4	0.4	3835	3763
5315-01	Hudson River	Poughkeepsie, NY	10/8/2019	2,043,354	1.23E+09	88.1	74.3	25.7	3.8	95.2	0.3	0.6	0.4	3203	3145
4981-01	Kansas River	DeSoto, KS	6/3/2019	2,358,286	1.41E+09	89.5	77.7	22.3	2.6	96.2	0.4	0.5	0.5	3332	3281
5036-01	Kansas River	DeSoto, KS	6/26/2019	1,431,519	8.59E+08	82.9	74.4	25.7	1.4	97.4	0.1	0.3	0.3	2540	2510
5075-01	Kansas River	DeSoto, KS	7/16/2019	3,158,582	1.90E+09	93.1	63.7	36.3	1.9	97.7	0.4	0.4	0.3	3956	3946
5022-01	Mississippi River	Hastings, MN	6/18/2019	1,162,448	6.97E+08	95.7	71.1	28.9	6.6	92.4	0.3	0.5	0.4	2422	2413
5029-01	Mississippi River	Hastings, MN	6/25/2019	2,318,197	1.39E+09	92.0	74.1	25.9	3.4	95.2	0.1	0.4	0.5	3599	3498
5079-01	Mississippi River	Hastings, MN	7/17/2019	1,430,960	8.59E+08	88.9	72.4	27.6	11.1	87.9	0.1	0.2	0.1	2640	2627
5105-01	Mississippi River	Hastings, MN	7/24/2019	1,263,942	7.58E+08	92.1	73.9	26.1	7.6	91.2	0.4	0.4	0.3	2454	2436
5105-02	Mississippi River	Hastings, MN	7/24/2019	2,362,441	1.42E+09	92.8	73.4	26.7	4.3	94.6	0.2	0.3	0.5	3600	3525
5145-01	Mississippi River	Hastings, MN	8/6/2019	3,512,517	2.11E+09	89.5	75.9	24.1	8.6	90.3	0.4	0.6	0.6	3933	3852
5323-01	Mississippi River	Hastings, MN	10/23/2019	1,377,252	8.26E+08	95.7	67.1	32.9	7.5	91.6	0.1	0.4	0.3	2546	2539
4986-01	Missouri River	Hermann, MO	6/4/2019	2,807,897	1.68E+09	89.5	77.3	22.7	3.1	95.9	0.2	0.4	0.3	3668	3619
5052-01	Missouri River	Hermann, MO	7/9/2019	1,398,940	8.39E+08	90.4	73.7	26.3	8.6	90.1	0.2	0.2	0.2	2716	2672
5095-01	Missouri River	Hermann, MO	7/23/2019	2,027,525	1.22E+09	92.6	74.6	25.4	7.1	91.6	0.2	0.2	0.2	3375	3293
5147-01	Missouri River	Hermann, MO	8/6/2019	1,414,440	8.49E+08	91.3	77.0	23.0	5.2	93.2	0.1	0.2	0.2	2655	2618
5302-01	Missouri River	Hermann, MO	10/1/2019	1,158,169	6.95E+08	94.8	70.3	29.8	2.2	96.7	0.1	0.3	0.3	2668	2637
5302-02	Missouri River	Hermann, MO	10/1/2019	901,365	5.41E+08	93.2	69.2	30.8	2.6	96.3	0.1	0.2	0.1	2328	2310
5012-01	Ohio River	Cannelton, IN	6/12/2019	2,301,203	1.38E+09	87.0	75.1	24.9	4.3	94.7	0.2	0.3	0.3	3274	3231
5153-01	Ohio River	Cannelton, IN	8/7/2019	1,227,778	7.37E+08	90.9	76.8	23.2	6.3	92.9	0.2	0.4	0.4	2303	2301
5313-01	Sacramento River	Freeport, CA	10/9/2019	1,803,692	1.08E+09	95.8	69.9	30.1	4.0	95.2	0.2	0.5	0.4	3154	3134
4988-01	Susquehanna River	Conowingo, MD	6/4/2019	2,372,956	1.42E+09	94.0	71.9	28.1	3.1	96.2	0.2	0.3	0.3	3617	3578
5154-01	Susquehanna River	Conowingo, MD	8/7/2019	1,956,465	1.17E+09	92.2	72.9	27.1	2.9	96.2	0.1	0.2	0.2	3451	3422
5310-01	Susquehanna River	Conowingo, MD	10/8/2019	1,606,502	9.64E+08	89.7	77.2	22.8	6.5	92.3	0.2	0.4	0.5	2886	2866
5310-02	Susquehanna River	Conowingo, MD	10/8/2019	1,429,660	8.58E+08	89.0	79.7	20.3	8.5	90.3	0.1	0.2	0.2	2497	2485
5021-01	Trinity River	Dallas, TX	6/18/2019	1,809,665	1.09E+09	94.6	75.7	24.3	7.1	91.4	0.0	0.2	0.1	3108	3063
5050-01	Trinity River	Dallas, TX	7/9/2019	2,024,985	1.21E+09	92.2	75.7	24.4	5.3	93.3	0.2	0.6	0.4	3281	3217
5096-01	Trinity River	Dallas, TX	7/23/2019	1,801,674	1.08E+09	93.5	73.8	26.2	3.6	95.1	0.1	0.6	0.4	3214	3168
5220-01	Trinity River	Dallas, TX	8/21/2019	2,280,022	1.37E+09	94.1	70.7	29.3	3.7	95.4	0.3	0.5	0.5	3692	3650
5220-02	Trinity River	Dallas, TX	8/21/2019	2,362,498	1.42E+09	95.8	68.9	31.1	4.1	95	0.3	0.5	0.4	3821	3770
5243-01	Trinity River	Dallas, TX	9/4/2019	2,066,899	1.24E+09	94.5	68.5	31.5	3.8	95.4	0.1	0.3	0.4	3526	3489
5006-01	Willamette River	Portland, OR	6/11/2019	1,915,663	1.15E+09	95.6	71.4	28.6	3.9	95.5	0.1	0.3	0.3	2976	2966
5049-01	Willamette River	Portland, OR	7/8/2019	1,892,247	1.14E+09	94.4	71.3	28.7	5.1	94.2	0.2	0.5	0.4	3287	3274
5146-01	Willamette River	Portland, OR	8/6/2019	1,646,360	9.88E+08	95.1	73.3	26.7	6.2	92.8	0.1	0.2	0.3	2938	2925
5211-01	Willamette River	Portland, OR	8/20/2019	2,121,095	1.27E+09	95.9	64.9	35.1	3.6	95.8	0.1	0.2	0.1	3327	3298
5257-01	Willamette River	Portland, OR	9/10/2019	2,064,569	1.24E+09	96.1	75.4	24.6	4.2	94.8	0.3	0.3	0.3	3308	3279
Averages	na	na	na	1,934,935	1.16E+09	92.5	70.5	29.5	4.0	95.1	0.2	0.4	0.3	3150	3121

Supplementary Table S2. Cyanobacteria *mcyE* sequencing information for microbial communities in U.S. rivers in 2019.

Sample ID	River	Month of Sample	Total Pairs of Sequences	Amplicon Sequence Variants detected
5104-01	Connecticut River	July	217465	13
5155-01	Connecticut River	August	277208	15
5155-02	Connecticut River	August	241133	16
5218-01	Connecticut River	August	189585	11
5244-01	Connecticut River	Sept	172290	10
5321-02	Delaware River	October	213600	11
5117-01	Hudson River	July	189592	12
5226-01	Hudson River	August	158846	11
5315-01	Hudson River	October	104601	17
5022-01	Mississippi River	June	163132	21
5029-01	Mississippi River	June	278359	21
5079-01	Mississippi River	July	1246	8
5105-01	Mississippi River	July	214032	32
5105-02	Mississippi River	July	229985	34
5145-01	Mississippi River	August	171239	31
5323-01	Mississippi River	October	177031	24
5095-01	Missouri River	July	967	8
5147-01	Missouri River	August	212596	26
5302-01	Missouri River	October	239984	22
5302-02	Missouri River	October	175699	19
4988-01	Susquehanna River	June	214423	10
5021-01	Trinity River	June	163048	20
5050-01	Trinity River	July	976	6
5243-01	Trinity River	Sept	184178	10
5146-01	Willamette River	August	183055	12
5211-01	Willamette River	August	183490	14
4996-01	Chattahoochee River	June		
5046-01	Chattahoochee River	July		
5197-01	Chattahoochee River	August		
5260-01	Chattahoochee River	Sept		
5300-01	Chattahoochee River	Sept		
5008-01	Connecticut River	June		
5038-01	Connecticut River	June		
5058-01	Connecticut River	July		
5314-01	Connecticut River	October		
5025-01	Delaware River	June		
5051-01	Delaware River	July		
5142-01	Delaware River	August		
5321-01	Delaware River	October		
5030-01	Hudson River	June		
5030-02	Hudson River	June		
4981-01	Kansas River	June	No detectable microcystin *mcyE* gene	
5036-01	Kansas River	June		
5075-01	Kansas River	July		
4986-01	Missouri River	June		
5052-01	Missouri River	July		
5012-01	Ohio River	June		
5153-01	Ohio River	August		
5313-01	Sacramento River	October		
5154-01	Susquehanna River	August		
5310-01	Susquehanna River	October		
5310-02	Susquehanna River	October		
5096-01	Trinity River	July		
5220-01	Trinity River	August		
5220-02	Trinity River	August		
5006-01	Willamette River	June		
5049-01	Willamette River	July		
5257-01	Willamette River	Sept		

Supplementary Table S3. Environmental data collected for U.S. river samples. All data are available through the National Water Information System (http://dx.doi.org/10.5066/F7P55KJN). U.S. Geological Survey Station Identification Numbers are as follows: Chattahoochee River: 02338000; Connecticut River: 01184000; Delaware River: 01463500; Hudson River: 01372043; Kansas River: 06892350; Mississippi River: 05331580; Missouri River: 06934500; Ohio River: 03303280; Sacramento River: 11447650; Susquehanna River: 01578310; Trinity River: 08057410; and Willamette River: 14211720.

Sample ID*	River	Date	Temperature, water, degrees Celsius	Dissolved oxygen, water, unfiltered, milligrams per liter	pH, water, unfiltered, field, standard units	Carbon dioxide, water, unfiltered, milligrams per liter	Total nitrogen [nitrate + nitrite + ammonia + organic-N], water, unfiltered, milligrams per liter	Nitrite, water, filtered, milligrams per liter as nitrogen	Nitrate, water, filtered, milligrams per liter as nitrogen	Phosphorus, water, unfiltered, milligrams per liter as phosphorus	Sulfate, water, filtered, milligrams per liter	Ammonia (NH_3 + NH_4^+), water, filtered, milligrams per liter as NH_4	Discharge (cubic feet per second)**	Turbidity NTRU**
4996-01	Chattahoochee River	6/5/2019	22.4	7.4	7.1	3.6	2.8	0.004	2.68	0.06	7.49	0.018	NA	6.7
5046-01	Chattahoochee River	7/2/2019	25.4	7.3	7.1	3.6	3	0.015	2.52	0.12	7.65	0.069	NA	6.4
5197-01	Chattahoochee River	8/19/2019	26.4	6.9	7.2	3.6	3.6	0.017	3.29	0.05	9.19	0.048	NA	6.5
5260-01	Chattahoochee River	9/11/2019	24.6	7.2	7.2	2.9	4.2	0.008	3.73	0.09	9.52	0.025	NA	13
5300-01	Chattahoochee River	9/30/2019	22.7	7.7	7.2	2.8	3.4	0.014	3.15	0.06	7.91	0.024	NA	5.2
5008-01	Connecticut River	6/12/2019	19.6	8.9	7.9	0.7	0.49	0.003	0.24	0.021	4.81	0.062	15100	<2
5038-01	Connecticut River	6/26/2019	21.3	8.5	7.5	2	0.56	0.005	0.24	0.032	4.9	0.11	12600	3.6
5058-01	Connecticut River	7/10/2019	25.8	8.3	7.6	1.7	0.59	0.005	0.349	0.029	6.1	0.075	10700	4
5104-01	Connecticut River	7/24/2019	24.5	7.6	7.6	1.6	0.54	0.007	0.303	0.033	5.84	0.052	16400	3.6
5155-01 & 5155-02	Connecticut River	8/7/2019	25.9	7.9	7.4	2.6	0.68	0.01	0.338	0.026	7.29	0.097	2940	<2
5218-01	Connecticut River	8/21/2019	26.8	7.7	7.8	1.1	0.53	0.006	0.297	0.016	6.86	0.041	6350	<2
5244-01	Connecticut River	9/4/2019	22.9	8.2	7.4	2.8	0.64	0.008	0.315	0.031	7.95	0.12	6630	2
5314-01	Connecticut River	10/9/2019	15.9	9.3	7.4	3.6	0.59	0.005	0.263	0.04	6.26	0.126	12600	2.3
5025-01	Delaware River	6/20/2019	19.4	8.7	7.4	3.2	1.2	0.006	0.741	0.098	9.01	0.039	23100	15
5051-01	Delaware River	7/9/2019	24.3	8.1	7.6	2.6	1.5	0.008	1.02	0.091	12.7	<0.013	11300	6.9
5142-01	Delaware River	8/5/2019	27.1	8.4	8.3	0.7	1.6	0.01	1.3	0.086	15.6	<0.013	5760	2.8
5321-01 & 5321-02	Delaware River	10/21/2019	11.7	10.7	7.1	5.4	1.1	0.005	0.788	0.071	10.3	0.031	7430	5.6
5030-01 & 5030-02	Hudson River	6/25/2019	21.4	8.9	7.9	0.9	0.67	0.012	0.379	0.052	8.85	0.016	NA	11
5117-01	Hudson River	7/24/2019	26.9	6.8	7.6	3	0.58	0.012	0.293	0.059	8.08	0.027	257000	13
5226-01	Hudson River	8/26/2019	27.4	6.4	7.7	3.2	0.59	0.004	0.315	0.043	13.7	0.026	NA	3.2
5315-01	Hudson River	10/8/2019	20.1	7.7	7.5	4.9	0.74	0.005	0.437	0.056	16	<0.013	NA	7.5
4981-01	Kansas River	6/3/2019	19.2	8.9	7.7	5.3	2.6	0.011	1.56	0.84	49.5	0.02	61300	140
5036-01	Kansas River	6/26/2019	23.1	7.5	8	1.8	4	0.041	1.59	0.97	53.5	<0.013	41000	470
5075-01	Kansas River	7/16/2019	24.6	8	7.8	4.2	2.3	0.009	1.5	0.58	65	<0.013	45700	80
5022-01	Mississippi River	6/18/2019	21.4	8.2	8	3.8	3.8	0.057	2.92	0.12	111	0.026	NA	22
5029-01	Mississippi River	6/25/2019	20.3	7.9	8	3.9	4.6	0.064	3.87	0.16	90.2	0.038	NA	34
5079-01	Mississippi River	7/17/2019	25.8	7.2	8.1	3.3	4.1	0.042	3.2	0.19	90.8	0.036	NA	31
5105-01 & 5105-02	Mississippi River	7/24/2019	24.5	7.4	8.1	3.4	3.7	0.039	3.04	0.22	84.2	<0.013	NA	36
5145-01	Mississippi River	8/6/2019	25.6	7.9	8.2	2.6	2.7	0.021	1.82	0.16	106	0.021	NA	23
5323-01	Mississippi River	10/23/2019	8.8	10.9	8.2	2.8	3.5	0.01	2.77	0.09	75.3	0.013	NA	17
4986-01	Missouri River	6/4/2019	21.8	5.6	7.5	6.7	2.5	0.073	1.38	0.562	49.1	0.046	383000	240
5052-01	Missouri River	7/9/2019	27.3	6.1	7.8	4.6	4	0.023	1.39	0.477	108	<0.013	268000	130
5095-01	Missouri River	7/23/2019	28.3	5.7	7.8	4.8	2	0.014	1.18	0.476	120	0.019	243000	85
5147-01	Missouri River	8/6/2019	26.8	5.7	7.8	5.6	1.9	0.013	0.934	0.386	128	<0.013	198000	96
5302-01 & 5302-02	Missouri River	10/1/2019	23.3	5.3	7.6	7.3	2.5	0.021	0.702	0.954	113	<0.013	241000	NA
5012-01	Ohio River	6/12/2019	22.9	7.1	7.5	5.2	1.6	0.005	1.05	0.261	44.2	<0.013	174000	70
5153-01	Ohio River	8/7/2019	28.6	6.7	7.8	2.2	1.2	0.021	0.973	0.067	52	0.028	44700	NA
5313-01	Sacramento River	10/9/2019	16.1	9.4	7.6	2.1	0.18	<0.001	<0.04	0.03	2.88	<0.013	8940	4.4
4988-01	Susquehanna River	6/4/2019	21.3	8.2	7.5	2.8	1.4	0.011	1.08	0.072	18.6	0.076	74100	28
5154-01	Susquehanna River	8/7/2019	30.4	7.1	7.5	3.6	1.4	0.055	0.941	0.029	32.1	0.077	6340	5.3
5310-01 & 5310-02	Susquehanna River	10/8/2019	23.8	8.7	7.6	3.5	1.3	0.14	0.881	0.027	46.5	0.079	4280	5.3
5021-01	Trinity River	6/18/2019	24.8	5.5	7.8	2.6	1.4	0.021	0.661	0.15	37.5	0.107	18200	61
5050-01	Trinity River	7/9/2019	28.2	7.2	7.7	4.4	6.6	0.013	0.831	0.15	33.8	0.045	7360	40
5096-01	Trinity River	7/23/2019	29	6.2	7.4	10	8.6	0.113	6.92	0.59	71.3	0.354	1050	38
5220-01 & 5220-02	Trinity River	8/20/2019	30.6	6	7.2	15	13	0.266	10.8	0.88	89.5	0.688	499	18
5243-01	Trinity River	9/4/2019	29.6	6.3	7.1	16	13	0.076	10.2	0.93	83.2	0.176	558	17
5006-01	Willamette River	6/11/2019	18.1	10.1	7.3	2.4	0.4	0.006	0.214	0.04	3.32	0.067	6050	2.3
5049-01	Willamette River	7/8/2019	20.6	8.6	7.1	4.5	0.67	0.017	0.361	0.04	4.37	0.103	26500	4
5146-01	Willamette River	8/6/2019	23.8	8.2	7.3	3.1	0.59	0.015	0.359	0.03	4.66	0.073	24600	2.8
5211-01	Willamette River	8/20/2019	22	8.5	7.4	2.5	0.6	0.013	0.366	0.05	4.5	0.101	12700	2.6
5257-01	Willamette River	9/10/2019	21.1	8.3	6.9	6.2	0.53	0.012	0.301	0.06	4.42	0.114	12900	2.8

NA = not available
< = less than (below detection)
*samples ending in -02 are concurrent replicates
**Data incomplete and thus not included in CCA

Supplementary Table S4. Trophic status of samples from U.S. rivers in this study.

ID*	River	Date of Sample	Chlorophyll a (µg/L)	Total nitrogen (µg/L)	Total phosphorus (µg/L)	Trophic category estimation**
4996-01	Chattahoochee River	6/5/2019	4.8	2800	60	Eutrophic
5046-01	Chattahoochee River	7/2/2019	1.2	3000	120	Eutrophic
5197-01	Chattahoochee River	8/19/2019	1	3600	50	Eutrophic
5260-01	Chattahoochee River	9/11/2019	2.1	4200	90	Eutrophic
5300-01	Chattahoochee River	9/30/2019	1.1	3400	60	Eutrophic
5008-01	Connecticut River	6/12/2019	0.9	490	21	Mesotrophic
5038-01	Connecticut River	6/26/2019	0.7	560	32	Mesotrophic
5058-01	Connecticut River	7/10/2019	3	590	29	Mesotrophic
5104-01	Connecticut River	7/24/2019	2.1	540	33	Mesotrophic
5155-01	Connecticut River	8/7/2019	1.6	680	26	Mesotrophic
5218-01	Connecticut River	8/21/2019	1.4	530	16	Mesotrophic
5244-01	Connecticut River	9/4/2019	1.6	640	31	Mesotrophic
5314-01	Connecticut River	10/9/2019	2.7	590	40	Mesotrophic
5025-01	Delaware River	6/20/2019	5.2	1200	98	Eutrophic
5051-01	Delaware River	7/9/2019	3	1500	91	Eutrophic
5142-01	Delaware River	8/5/2019	1.7	1600	86	Eutrophic
5321-01	Delaware River	10/21/2019	1.7	1100	71	Meso-Eutrophic
5030-01	Hudson River	6/25/2019	16.9	670	52	Meso-Eutrophic
5117-01	Hudson River	7/24/2019	3.4	580	59	Mesotrophic
5226-01	Hudson River	8/26/2019	3	590	43	Mesotrophic
5315-01	Hudson River	10/8/2019	n/a	740	56	Eutrophic
4981-01	Kansas River	6/3/2019	2.3	2600	840	Hypertrophic
5036-01	Kansas River	6/26/2019	3.4	4000	970	Hypertrophic
5075-01	Kansas River	7/16/2019	4.2	2300	580	Hypertrophic
5022-01	Mississippi River	6/18/2019	20.2	3800	120	Hypertrophic
5029-01	Mississippi River	6/25/2019	13.2	4600	160	Hypertrophic
5079-01	Mississippi River	7/17/2019	16	4100	190	Hypertrophic
5105-01	Mississippi River	7/24/2019	12.6	3700	220	Hypertrophic
5145-01	Mississippi River	8/6/2019	5.9	2700	160	Hypertrophic
5323-01	Mississippi River	10/23/2019	10.3	3500	90	Hypertrophic
4986-01	Missouri River	6/4/2019	n/a	2500	562	Hypertrophic
5052-01	Missouri River	7/9/2019	12.9	4000	477	Hypertrophic
5095-01	Missouri River	7/23/2019	11.9	2000	476	Hypertrophic
5147-01	Missouri River	8/6/2019	n/a	1900	386	Hypertrophic
5302-01	Missouri River	10/1/2019	8.4	2500	954	Hypertrophic
5012-01	Ohio River	6/12/2019	2.9	1600	261	Hypertrophic
5153-01	Ohio River	8/7/2019	3.4	1200	67	Eutrophic
5313-01	Sacramento River	10/9/2019	1.3	180	30	Oligotrophic
4988-01	Susquehanna River	6/4/2019	7.1	1400	72	Eutrophic
5154-01	Susquehanna River	8/7/2019	5.1	1400	29	Eutrophic
5310-01	Susquehanna River	10/8/2019	2	1300	27	Eutrophic
5021-01	Trinity River	6/18/2019	11.1	1400	150	Hypertrophic
5050-01	Trinity River	7/9/2019	11.7	6600	150	Hypertrophic
5096-01	Trinity River	7/23/2019	14.5	8600	590	Hypertrophic
5220-01	Trinity River	8/20/2019	19.9	13000	880	Hypertrophic
5243-01	Trinity River	9/4/2019	15.9	13000	930	Hypertrophic
5006-01	Willamette River	6/11/2019	6.9	400	40	Meso-Eutrophic
5049-01	Willamette River	7/8/2019	5.4	670	40	Meso-Eutrophic
5146-01	Willamette River	8/6/2019	12.1	590	30	Mesotrophic
5211-01	Willamette River	8/20/2019	4.9	600	50	Meso-Eutrophic
5257-01	Willamette River	9/10/2019	2.8	530	60	Meso-Eutrophic

*Replicate samples (ending in -02) are not shown
**See Tanvir, Hu, Zhang, & Lu, 2021 for estimation criteria

Supplementary Table S5. Primers used in this study.

Gene	Size (bp)	Primers	Sequence (5'-3')	Purpose	Reference
mcyE	472	HEPF	TTTGGGGTTAACTTTTTTGGGCATAGTC	*mcyE* /HEP amplicon sequencing	Jungblut & Neilan 2006
		HEPR	AATTCTTGAGGCTGTAAATCGGGTTT		
Cyanobacteria 16S rRNA	269	CYAN108F	ACGGGTGAGTAACRCGTRA	qPCR	Nubel et al. 1997
		CYAN377R	CCATGGCGGAAAATTCCCC		
Microcystis 16S rRNA	248	MIC209F	ATGTGCCGCGAGGTGAAACCTAAT		Neilan et al. 1997
		MIC409R	TTACAATCCAAAGACCTTCCTCCC		
